# Identification of three subtypes of triple-negative breast cancer with potential therapeutic implications

**DOI:** 10.1186/s13058-019-1148-6

**Published:** 2019-05-17

**Authors:** Pascal Jézéquel, Olivier Kerdraon, Hubert Hondermarck, Catherine Guérin-Charbonnel, Hamza Lasla, Wilfried Gouraud, Jean-Luc Canon, Andrea Gombos, Florence Dalenc, Suzette Delaloge, Jérôme Lemonnier, Delphine Loussouarn, Véronique Verrièle, Mario Campone

**Affiliations:** 1 Département de Biopathologie, Unité Mixte de Génomique du Cancer, Institut de Cancérologie de l’Ouest – site René Gauducheau, Bd Jacques Monod, 44805 Saint Herblain Cedex, France; 2 Unité de Bioinfomique, Institut de Cancérologie de l’Ouest, Bd Jacques Monod, 44805 Saint Herblain Cedex, France; 3 CRCINA, UMR 1232 INSERM, Université de Nantes, Université d’Angers, Institut de Recherche en Santé-Université de Nantes, 8 Quai Moncousu, BP 70721, 44007 Nantes Cedex 1, France; 4 Laboratoire d’Anatomie et Cytologie Pathologiques, Institut de Cancérologie de l’Ouest, Bd Jacques Monod, 44805 Saint Herblain Cedex, France; 5 School of Biomedical Sciences and Pharmacy, Hunter Medical Research Institute, University of Newcastle, Callaghan, NSW 2308 Australia; 6 CRCINA, INSERM, CNRS, Université de Nantes, Université d’Angers, Institut de Recherche en Santé-Université de Nantes, 8 Quai Moncousu, BP 70721, 44007 Nantes Cedex 1, France; 7 Oncologie-Hématologie, Grand Hôpital de Charleroi, 3 Grand’Rue, 6000 Charleroi, Belgium; 8 Oncologie Médicale, Institut Jules Bordet, 121 Bd de Waterloo, 1000 Bruxelles, Belgium; 9 Oncologie Médicale, IUCT-Oncopole, 1 Av Irène Joliot-Curie, 31100 Toulouse, France; 10 Oncologie Médicale, Gustave Roussy, 114 rue Edouard Vaillant, 94800 Villejuif, France; 11 UCBG, R&D UNICANCER, Fédération Nationale des Centres de Lutte Contre le Cancer, 101 rue de Tolbiac, 75013 Paris Cedex 13, France; 12 Départment d’Anatomie et Cytologie Pathologiques, Centre Hospitalo-Universitaire, 1 place Alexis Ricordeau, 44093 Nantes, France; 13 Oncologie Médicale, Institut de Cancérologie de l’Ouest, René Gauducheau, Bd Jacques Monod, 44805 Saint Herblain Cedex, France

**Keywords:** Breast cancer, Triple-negative, Transcriptomics, Molecular subtypes, Immunome, Tertiary lymphoid structures, Neurogenesis

## Abstract

**Background:**

Heterogeneity and lack of targeted therapies represent the two main impediments to precision treatment of triple-negative breast cancer (TNBC), and therefore, molecular subtyping and identification of therapeutic pathways are required to optimize medical care. The aim of the present study was to define robust TNBC subtypes with clinical relevance.

**Methods:**

Gene expression profiling by means of DNA chips was conducted in an internal TNBC cohort composed of 238 patients. In addition, external data (*n* = 257), obtained by using the same DNA chip, were used for validation. Fuzzy clustering was followed by functional annotation of the clusters. Immunohistochemistry was used to confirm transcriptomics results: CD138 and CD20 were used to test for plasma cell and B lymphocyte infiltrations, respectively; MECA79 and CD31 for tertiary lymphoid structures; and UCHL1/PGP9.5 and S100 for neurogenesis.

**Results:**

We identified three molecular clusters within TNBC: one molecular apocrine (C1) and two basal-like-enriched (C2 and C3). C2 presented pro-tumorigenic immune response (immune suppressive), high neurogenesis (nerve infiltration), and high biological aggressiveness. In contrast, C3 exhibited adaptive immune response associated with complete B cell differentiation that occurs in tertiary lymphoid structures, and immune checkpoint upregulation. External cohort subtyping by means of the same approach proved the robustness of these results. Furthermore, plasma cell and B lymphocyte infiltrates, tertiary lymphoid structures, and neurogenesis were validated at the protein levels by means of histological evaluation and immunohistochemistry.

**Conclusion:**

Our work showed that TNBC can be subcategorized in three different subtypes characterized by marked biological features, some of which could be targeted by specific therapies.

**Electronic supplementary material:**

The online version of this article (10.1186/s13058-019-1148-6) contains supplementary material, which is available to authorized users.

## Background

Triple-negative breast cancer (TNBC) represents 12 to 17% of primary breast cancer and is the most aggressive and deadly breast cancer subtype [1]. Furthermore, heterogeneity and lack of targeted therapies represent the two main issues for precision treatment of TNBC patients. Molecular subtyping and identification of therapeutic pathways are therefore required to optimize medical care of these patients.

Recent works based on different approaches identified various numbers of TNBC clusters [2, 3]. Six molecular clusters were found in two in silico studies. In the first study, basal-like 1, basal-like 2, immunomodulatory, mesenchymal, mesenchymal stem-like, and luminal androgen receptor clusters were described [3]. In the second study, clusters were named immunity 1, immunity 2, proliferation/DNA damage, androgen receptor-like, matrix/invasion 1, and matrix/invasion 2 [4]. TNBC status was based on bimodal filtering of estrogen receptor (ER), progesterone receptor (PR), and human epidermal growth factor receptor 2 (HER2) gene expressions in the first study, and bimodal filtering of estrogen receptor 1 and Erb-B2 receptor tyrosine kinase 2 genes, and median value of progesterone receptor gene in the second study. In addition, four clusters (luminal androgen receptor, mesenchymal, basal-like immunosuppressed, and basal-like immune-activated) and three clusters (molecular apocrine, basal-like-enriched with low immune response and high M2/M1 macrophages ratio, and basal-like-enriched with high immune response and low M2/M1 macrophage ratio) were found in two internal immunohistochemistry (IHC)-typed TNBC studies, respectively [5, 6]. Although different number of clusters was found, three clusters seem to be present in each of these works: molecular apocrine and two basal-like-enriched clusters with opposite immune response (pro-tumorigenic and anti-tumorigenic). Today, TNBC subtyping still needs to be refined.

In the present study, an unsupervised analysis was conducted on an internal training cohort composed of 238 TNBC tumors. Fuzzy clustering was followed by functional annotation of the clusters, and we intensively focused on exploring the nature of immune response between the two basal-like-enriched clusters. Together, the results identified three TNBC clusters: one molecular apocrine (C1) and two basal-like-enriched, of which one with pro-tumorigenic immune response (immune suppressive), high neurogenesis activity and high biological aggressiveness (C2), and the other with adaptive immune response associated with complete B cell differentiation and immune checkpoint upregulation (C3).

## Materials and methods

### Patients

Internal cohort was composed of 238 TNBC patients. One hundred seven patients were part of a previous study, and 131 other TNBC patients were enrolled in the UNICANCER PACS08 (NCT00630032) adjuvant multicenter trial described elsewhere [6, 7]. This last study was approved by a French Ethic Committee (CPP Ouest V, CHU Pontchaillou, Rennes, France; reference number: 07/09-626).

An external cohort composed of publicly available TNBC patients with available tumor genomic data was built. To avoid cross-platform normalization issues, we exclusively looked for Affymetrix® genomic datasets in repositories such as Gene Expression Omnibus (GEO) and ArrayExpress, selecting those with a medium to large sample size [8, 9]. Non-TNBC data from these studies were also retrieved (Additional file 1).

### Tumor tissues

All tumor tissue samples were surgically collected and processed in two parts by a pathologist. The first part was fixed in 10% neutral buffered formalin for standard histological analysis and IHC. The second part was immediately dissected, snap-frozen in liquid nitrogen, and stored until RNA extraction.

### RNA extraction

Total RNA was prepared following standard protocols then treated with DNase I using the RNeasy column purification system (Qiagen, France). Assessment of RNA quality, integrity, and purity was done through a Bionalyser 2100 (Agilent Technologies, Palo Alto, CA). RNA samples were considered for further analysis only if they had distinct 28 S and 18 S ribosomal peaks.

### Gene expression profiling

Gene expression analysis was performed using Affymetrix® Human Genome U133 Plus 2.0 Arrays (Affymetrix®, Santa Clara, CA) measuring over 54,000 transcripts representing over 20,000 genes. cRNA synthesis, labelling as well as chip hybridization, washing, and image scanning were performed according to the manufacturer’s protocol. Affymetrix® na35 probe set annotation was used.

### Bioinformatics

#### Internal and external data pre-processing

For both internal and external cohorts, raw data were MAS5-normalized in the Affymetrix® Expression Console (v1.3.1) and then log2-transformed. Genes were then median-centered and scaled in each cohort separately. All microarrays complied with quality criteria. Microarray and patient clinical data have been deposited in the GEO under the GSE83937 accession number. Five publicly available datasets were pooled for a total of 257 TNBC (Additional file 1).

#### Unsupervised analysis

To organize data into groups with the same underlying molecular characteristics, we performed clustering analysis, based on the 5% most variable probe sets (*n* = 1843; intersection of the 2 sets of most variable probes), by means of fuzzy clustering method (6).

#### Cluster functional annotation

To annotate the clusters, we used clinicopathologic characteristics, 54 gene-expression signatures (GES), Gene Ontology enrichment analysis (GOEA), and non-TNBC data. Depending on the nature of data, the following methods helped to explore differences among the clusters: one-way analysis of variance (ANOVA) with Tukey post hoc test, Fisher’s exact test, Cox regression model, Kaplan-Meier curves with log-rank test, and Pearson’s correlation coefficient.

#### Gene-expression signatures

Fifty-four GES were selected for functional annotation of breast cancer tumors. Twelve GES were used for breast cancer molecular subtyping: 4-TNBC, androgen receptor (AR), basal-like, tumor identity card (CIT), claudin-CD24, claudin-low, ER, ER-negative, ERBB2, molecular apocrine, PAM50, and TNBCtype. Eleven were used for immune response dissection: B-cell, Cell type Identification By Estimating Relative Subsets Of known RNA Transcripts (CIBERSORT) [*n* = 22] (http://cibersort.stanford.edu/), cytolytic activity (CYT), interferon (type I IFN), interleukin-8 (IL-8), M2/M1 macrophage ratios (M2/M1, M2/M1 [Becker]), MHC-1, MHC-2, STAT1, and T cell [10]). Three relate to microenvironment cells: epithelial cells, fibroblasts, and neurons (http://xcell.ucsf.edu/). Three were linked to metabolism evaluation: adipocytes, glycolysis, and iron (IRGS); and 22 to critical biological pathways in cancer: AKT, β-catenin, chromosomal instability (CIN), E2F3, EGFR, HOXA, mitochondrial oxidative phosphorylation (MITO/OXPHOS), MYC, p53, PIK3CA, perineural invasion (PNI), prolactin (PRL), proliferation, PTEN loss, RAS, reactive stroma, SRC, Stroma-CD10, TGFβ, VEGF, wound response, and YAP1-WWTR1. Finally, three prognostic GES were also used: 38-GES, van’t Veer 70-GES, and genomic grade index (GGI). Complete GES list, methods, and references are described in Additional file 2.

#### GOEA

Functional annotation of each cluster through GO biological process analysis was performed using the ToppGene web tool [11]. Two methods were used to select genes differentially expressed across the clusters. SAM method was performed to obtain lists of genes with significantly different expression between clusters (one versus one and one versus the others): genes for which all corresponding probe sets had a *q* value of 0% were retained. In addition to SAM method, expression of the 5% most variable probe sets was represented on a heatmap, with patients ordered according to the clusters; hierarchical clustering (centered Pearson correlation distance, Ward’s method) was performed on the probe sets in order to visually detect groups of genes with high expression patterns corresponding to specific cluster(s) of patients. Visually identified overexpressed gene sets in heatmap were designated by “H” followed by a number, which referred to the number of the corresponding cluster (1, 2, or 3). If sets of genes contained different subsets, this number was followed by “a,” “b,” or “c.”

#### Histological evaluations, tissue microarrays, and immunohistochemistry

Eighty-seven cases of archival formalin-fixed, paraffin-embedded tissues of the internal cohort were evaluated on tissue microarrays, containing a median of 3 replicate 0.6 mm cores per case. Tissue microarrays (TMA) construction was described elsewhere [6]. Furthermore, 42 full sections of these surgical specimens were available for histological evaluation. Immunohistochemistry was performed using the Ventana BenchMark Ultra platform (Ventana Medical Systems, Tucson, AZ). Details of the antigen retrieval technique and dilution of primary antibodies (CD20, CD21, CD138, MECA79, UCHL1/PGP9.5, and S100) are described in Additional file 3.

Tumor-infiltrating lymphocytes (TILs), especially CD138-positive plasma cells and CD20-positive B lymphocytes, were assessed according to recommendations of an international working group [12, 13]. The presence of tertiary lymphoid structure (TLS) and lymphoid clusters, with and without germinal centers, respectively, was evaluated in the 42 specimens with available full sections, due to their typical localization in the surrounding area of the tumors. Furthermore, the number of CD21-positive follicular dendritic network was assessed by counting positive structures by IHC on a representative full section per tumor [14]. The presence of high endothelial venules (HEV) was assessed on full sections by counting the number of MECA79-positive vessels in five 400× magnification hotspots per tumor [15]. Cases were classified as nerve fibers positive versus nerve fibers negative using UCHL1/PGP9.5 (neuronal marker) and S100 (pan-specific Schwann cell marker). Pathologists were blinded for TNBC cluster assignment.

#### Non-TNBC external data testing

Non-TNBC external data (*n* = 894) were used to refine non-basal-like TNBC cluster (Additional file 1).

#### Statistical analysis

We considered a two-sided *P* value of less than 5% to be statistically significant. For SAM method, 0% *q* values were retained. All statistical analyses and figures’ generations were performed using R [16] and the packages affy 1.50.0, amap 0.8.14, cluster 2.0.4, citbcmst 1.0.4, fpc 2.1.10, and samr 2.0.

## Results

### Unsupervised analysis

Principal component analysis (PCA) with the projection of the two internal TNBC cohorts onto the first principal plane showed a homogeneous distribution of PACS08 patients compared to TNBC patients included in a previous study, which rules out cohort and technical biases, and permits us to merge these data (Additional file 4). Fuzzy clustering separated TN tumors into three clusters, named C1 (*n* = 55; 23.1%), C2 (*n* = 98; 41.2%), and C3 (*n* = 85; 35.7%) (Additional file 5). Distributions of patients among the three clusters in the two IHC cohorts were similar to the one measured in the internal TNBC one (TMA, *P* = 0.8914; full section, *P* = 0.3362). This cohorts’ representativeness permits us to use these two IHC cohorts for marker validation.

### Cluster functional annotation

#### Clinicopathologic characteristics

Three clinicopathologic characteristics were differentially represented in function of clusters (Table 1). Patients belonging to C1 were older than C3 patients (Tukey, *P* = 0.0066). Histological grade and Nottingham prognostic index were higher in C2 compared to C1. Contrary to our previous work based on 107 patients of this larger TNBC cohort, no prognostic difference was found between the three clusters: overall survival, *P* = 0.54; metastasis-free survival, *P* = 0.64; and event-free survival (EFS), *P* = 0.41. EFS analysis conducted on internal and external cohorts showed no significant differences (*P* = 0.20 and 0.07, respectively); however, pooling both cohorts (*n* = 427) showed that C3 patients have a better prognosis compared to C2 patients (*P* = 0.0480) (Additional file 6).

**Table 1:** Clinicopathologic characteristics of the triple-negative studied tumors in function of cluster assignment

Variable	All (*n* = 238)	Cluster 1 (*n* = 55)	Cluster 2 (*n* = 98)	Cluster 3 (*n* = 85)	*P*
Age (years; mean ± sd)	54.6 ± 11.6	58.4 ± 11.4	54.4 ± 11.8	52.3 ± 11.0	0.0095
SBR grade					
1	3	2	0	1	0.0054
2	40	16	10	14	
3	195	37	88	70	
Tumor size (mm; mean ± sd)	24.3 ± 12.7	24.3 ± 15.1	25.1 ± 11.7	23.5 ± 12.2	0.69
Nodal status					
0	129	28	54	47	0.85
1	108	27	43	38	
NPI					
1	15	9	1	5	0.0075
2	160	31	71	58	
3	61	14	25	22	
Radiotherapy					
No	11	3	3	5	0.67
Yes	224	51	94	79	
Adjuvant therapy					
No	13	6	5	2	0.10
Yes	225	49	93	83	
Hormonotherapy					
No	220	51	92	77	0.94
Yes	14	3	5	6	
Metastasis					
No	184	41	75	68	0.74
Yes	54	14	23	17	

*sd* standard deviation, *SBR* Scarff Bloom Richardson, *NPI* Nottingham prognostic index

#### PCA results

First principal component (PC1) separated C1 tumors from C2 and C3 tumors, while second principal component (PC2) separated C2 from C3 (Additional file 7). The 20 probe sets of each of the two components with the highest absolute weights were selected. A total of 20 unique genes (15 for PC1 and five for PC2) could be identified and were retained for bibliographic analysis (Additional file 8). Eight PC1 genes were known to be linked to molecular apocrine subtype. Seven belonged to a set of genes upregulated in PIK3CA-mutated ERalpha-positive breast cancer cells. Association between high AR level and PIK3CA mutations was consistent with Lehmann’s work, which showed that mutations in *PIK3CA* exon 9 and 20 were more frequent in luminal androgen receptor TNBC subtype [17]. The five well-identified PC2 genes were specific of B cell lineage differentiation. In conclusion, PCA showed that the two biological key features that distinguished the three TNBC clusters were molecular apocrine phenotype for C1 compared to C2 and C3, and B cell lineage differentiation for C3 compared to C2.

### Gene-expression signatures

#### Molecular clustering

**Fig. 1:**
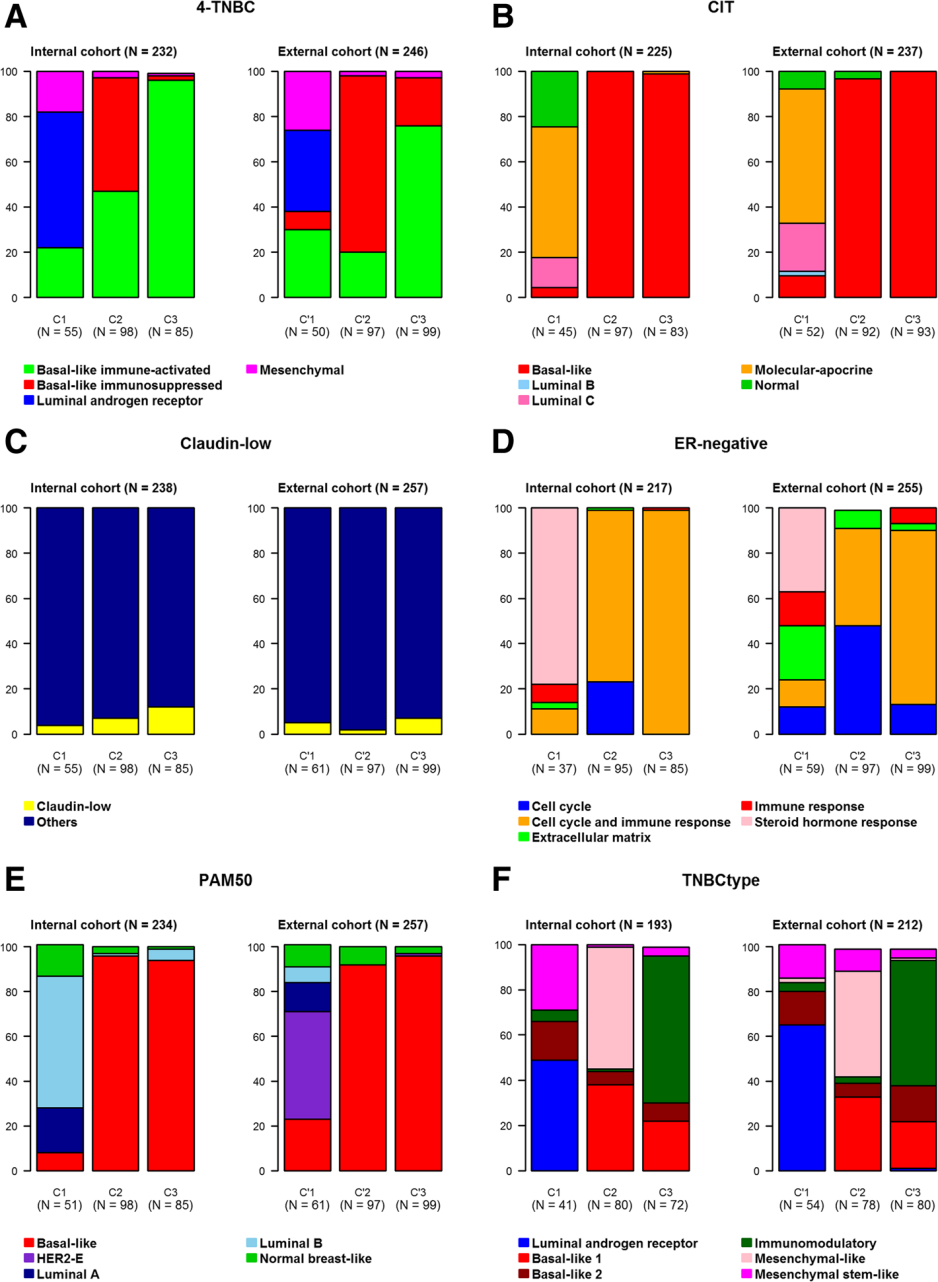
Subtype distributions of patients between the three clusters by means of categorical GES, for the internal (left) and external (right) TNBC cohorts. **a** 4-TNBC. **b** CIT. **c** Claudin-low. **d** ER-negative. **e** PAM50. **f** TNBCtype

**Fig. 2:**
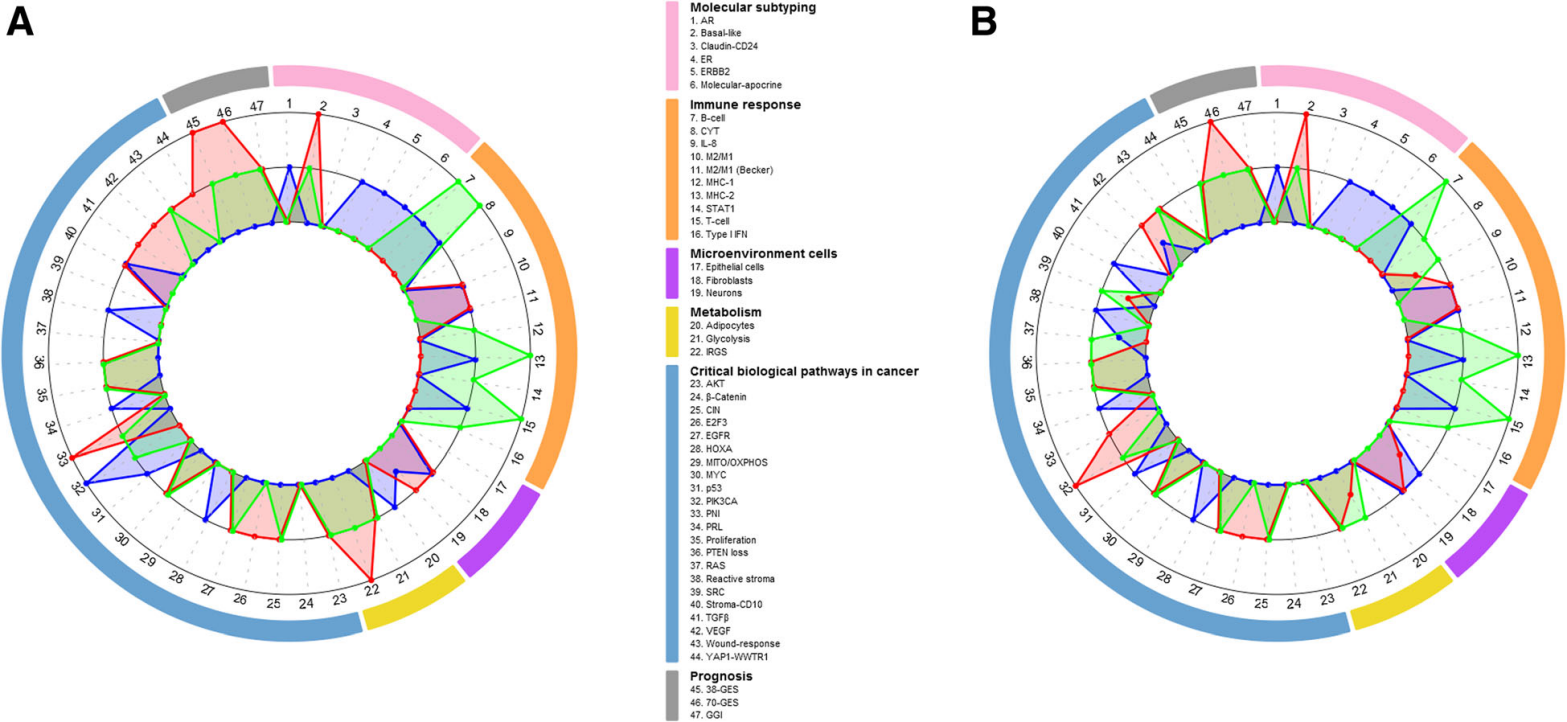
Molecular annotation of TNBC by means of continuous score GES in function of clusters. **a** Internal cohort. **b** External cohort. Differences in GES scores according to clusters (ANOVA results) are represented as a radar plot, where each of the 47 radii represents a GES. Black circles represent significantly different levels of expression from low (smallest circle) to high (largest circle). Expression level of each cluster is represented on the corresponding circle as a blue (C1, C’1), red (C2, C’2), or green (C3, C’3) dot. Dots located on same circles correspond to clusters with not significantly different expressions. Dots located on different circles correspond to clusters with significantly different expressions. Dots located in between black circles correspond to a cluster with expression level not significantly different from clusters whose dots are located on both near circles. This figure is an illustration of Additional files 10 and 21 statistical analyses

Various GES indicated that C1 clustered molecular apocrine (luminal androgen receptor) breast tumors (Figs. 1 and 2 and Additional files 9 and 10). Molecular apocrine subtype identification by means of GES was concordant with previous IHC results (AR, FOXA1) [6]. This subtype is known to be characterized by a molecular profile common to ER-positive breast cancer and an enrichment in ERBB2-positive tumors [18–21]. These two characteristics were demonstrated by means of ER and ER-negative GES, and ERBB2 GES, respectively.

Basal-like features were clearly displayed by C2 and C3 based on different GES (CIT, PAM50, 4-TNBC, basal-like) (Additional files 9 and 10).

#### Critical biological pathways in cancer

Numerous GES (CIN, EGFR, HOXA, MYC, p53, PNI, proliferation, wound response) showed that the biological aggressiveness of TNBC was high in C2 and C3 compared to C1 and more pronounced (i.e., mesenchymal phenotype) in C2 compared to C3 (E2F3, PNI, VEGF, YAP1-WWTR1) (Additional file 10).

#### Immune response

We focused on immune response in C2 and C3 because intratumoral immune response is known to play an important role in basal-like subtypes and has prognostic significance. Two immune GES, relative to M2/M1 macrophage ratios, displayed high scores in C2 compared to C3 (*P* <  0.0001), a finding which was confirmed by CIBERSORT analysis (Table 2). Since M2 macrophages are considered as immunosuppressive cells, this result underlined an immune pro-tumorigenic signal in C2 compared to C3.

**Table 2:** C2 vs C3 CIBERSORT analysis results

Immune cells	Tumorigenic effect in breast cancer^a^	*P*	C2 vs C3 differential expression
B cells memory	Pro-tumorigenic	0.0242	C2 > C3
B cells naïve	No effect	0.0798	C2 ≈ C3
Dendritic cells activated	Anti-tumorigenic	0.0177	C2 > C3
Dendritic cells resting	Pro-tumorigenic	0.4011	C2 ≈ C3
Eosinophils	Pro-tumorigenic	0.0243	C2 > C3
Macrophages M0	Pro-tumorigenic	< 0.0001	C2 > C3
Macrophages M1	Anti-tumorigenic	0.0005	C2 < C3
Macrophages M2	Pro-tumorigenic	< 0.0001	C2 > C3
Mast cells activated	Pro-tumorigenic	< 0.0001	C2 > C3
Mast cells resting	Anti-tumorigenic	0.0414	C2 < C3
Monocytes	Pro-tumorigenic	0.0169	C2 > C3
Neutrophils	Pro-tumorigenic	0.4571	C2 ≈ C3
NK cells activated	Anti-tumorigenic	0.5534	C2 ≈ C3
NK cells resting	Pro-tumorigenic	0.3776	C2 ≈ C3
Plasma cells	Anti-tumorigenic	< 0.0001	C2 < C3
T cells CD4 memory activated	Anti-tumorigenic	< 0.0001	C2 < C3
T cells CD4 memory resting	Pro-tumorigenic	0.0481	C2 > C3
T cells CD4 naive	Anti-tumorigenic	0.2901	C2 ≈ C3
T cells CD8	Anti-tumorigenic	0.0001	C2 < C3
T cells follicular helper	Anti-tumorigenic	0.2394	C2 ≈ C3
T cells gamma delta	Anti-tumorigenic	< 0.0001	C2 < C3
T cells regulatory	Anti-tumorigenic	0.4372	C2 ≈ C3

^a^Based on results from Gentles et al. [21] except for macrophages M1 [22]

High TGFβ GES scores confirmed C2 pro-tumorigenic status. Indeed, in late-stage tumors, TGFβ acts as a pro-tumorigenic cytokine produced by tumor cells and tumor-infiltrating immune cells [22].

All other immune GES (B-cell, IFN type I, CXCL8 [IL8], MHC-1, MHC-2, STAT1, T cell, and notably CYT GES based on the expression of *GZMA* [granzyme A] and *PRF1* [perforin 1]) showed high scores in C3, which suggested a pronounced adaptive immune response.

CIBERSORT analysis showed that 14 out of 22 immune cells were differentially distributed among C2 and C3 (Table 2). According to relationships between these hematopoietic subsets and survival in breast cancer, C2 tumors were highly infiltrated by seven immune cells associated with adverse outcomes (hereafter named “pro-tumorigenic” immune cells) [23]. On the contrary, C3 tumors were highly infiltrated by six immune cells associated with favorable outcomes (hereafter named “anti-tumorigenic” immune cells). Contrary to Gentles’ results and according to numerous studies, macrophages M1 were considered as anti-tumorigenic immune cells [24]. Out of 14 immune subsets, only dendritic cells activated were discordant with this interpretation.

In brief, two opposite immunophenotypes have been identified in basal-like TNBC; the first one characterized by immune cells known to stimulate breast cancer growth (pro-tumorigenic) and the second one by immune cells known to inhibit it (anti-tumorigenic). Furthermore, prognostic analyses based on immune continuous score GES in internal and external cohorts showed that C2 and C3 patients with high CYT, MHC-2, and STAT1 scores, reflecting an anti-tumorigenic immune response, have a better prognosis (Additional file 11).

#### Metabolism

Gene expression related to iron metabolism clearly decreased from C2 to C3 and from C3 to C1 (Additional file 10). Glycolysis seemed more elevated in C2 and C3 compared to C1. Considering biological knowledge, high iron metabolism and glycolysis are associated with aggressive tumors [25, 26].

#### Prognosis

Scores calculated according to the three prognostic GES were significantly lower in C1 compared to C2 and C3 (Additional file 10). The 70-GES prognostic score kinetics pattern separated the three clusters in the following order: C2 > C3 > C1. This finding was not corroborated by prognostic analysis. Therefore, we can only conclude that it more likely underlines biological aggressiveness of C2 and C3 tumors.

#### GOEA and neurogenesis

**Fig. 3:**
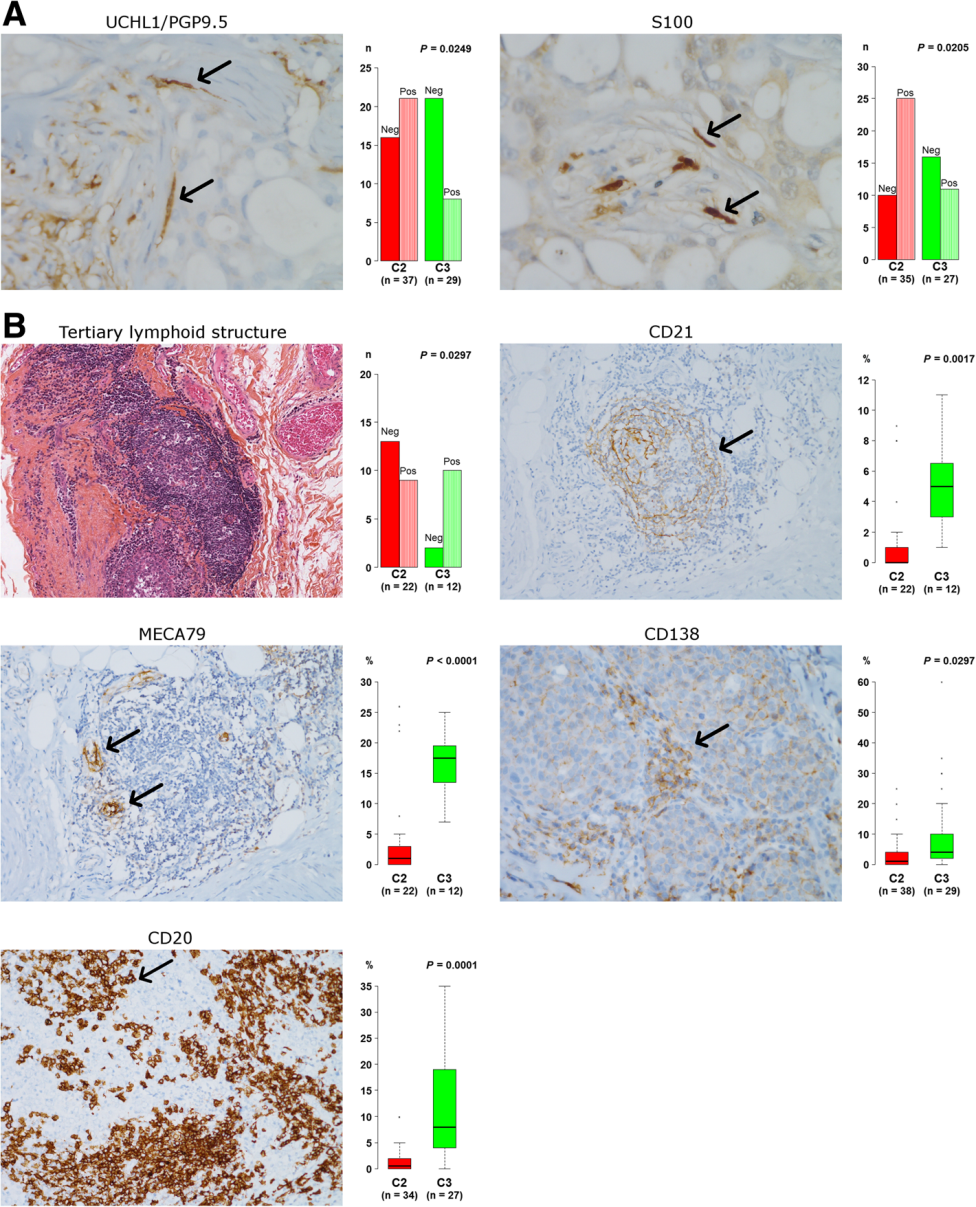
Immunohistochemistry and histological evaluations of neurogenesis and immune markers. **a** Detection of nerve fibers in C2 TNBC tumors. Nerve fibers in the tumoral stroma, with their typical spindled and wavy morphology, detected with IHC against the axonal marker UCHL1 (60×) and the schwannian marker S100 (60×). UCHL1 and S100 displayed a C2 > C3 profile. **b** Immune features of C3 TNBC tumors. Tertiary lymphoid structure (TLS) in the vicinity of invasive front of carcinoma, defined by the presence of a germinal center (hematoxylin and eosin staining) (10×), highlighted by follicular dendritic cells marker CD21 (20×). HEV, specialized blood vessels in lymphocytes recruitment, stained by MECA79 (20×), preferentially found in lymphocytes-rich regions of tumors. Plasma cell and B lymphocyte infiltrates were analyzed, respectively, with CD138 and CD20 stainings in the tumoral stroma (40×). Infiltrates were assessed according to recommendations of an international working group, by determining the area occupied by plasma cells or B lymphocytes over the total intratumoral stromal area. TLS, CD21, MECA79, CD138 and CD20 displayed a C3 > C2 profile. Arrows indicate cells expressing each marker (brown). Statistical plots on the right of each picture display the numbers (Pos: positive; Neg: negative) or percentages of marked cells in C2 compared to C3

Four clusters named H1, H2a, H2b, and H3 of highly expressed genes, belonging to C1, C2, and C3, were visually individualized from heatmap composed of the 5% most variable probe sets (*n* = 1843). Furthermore, eight gene lists of most differentially expressed genes between the three clusters were obtained by means of SAM. All these gene lists were submitted to the ToppGene web tool (Additional files 12 and 13). C1 was characterized by three luminal hallmarks (hormone metabolic process, lipid metabolic process, and oxidation-reduction process) on one hand, and angiogenesis and developmental processes, on the other hand. Mitotic cell cycle process, which is considered as a basal-like hallmark, was overrepresented in C2 and C3 compared to C1. Genes highly expressed in C2 vs C3 were notably linked to central nervous system development process, here considered as neurogenesis, extracellular matrix disassembly, and collagen metabolic process, i.e., invasion and progression. To our knowledge, this is the first time that high neurogenesis-related gene expression is linked to a breast cancer subtype. This finding was associated with UCHL1/PGP9.5 neuronal marker and S100 pan-specific Schwann cell marker enrichment in C2 compared to C3: *P* = 0.0249 and *P* = 0.0205, respectively (Fig. 3). Neuron GES corroborated this difference. Finally, high PNI scores in C2 (C2 > C3 > C1) could result from high nerve density in this subtype (Additional file 10). Further investigations are needed to unravel the role of nerve cells and de-differentiated Schwann cells in cancer progression and invasion [27, 28]. Immune response activation, and notably B cell activation, was preponderant in C3.

#### H3 witnesses the presence of tertiary lymphoid structures in close association with C3 TNBC subtype

Three gene subclusters (H3a, H3b, and H3c) could be distinguished in H3. GenomicScape was used to refine B lymphocyte subsets in these three gene subclusters [29]. H3c was strongly enriched in genes highly expressed in plasma cells (Additional file 14). On the contrary, H3b genes focused our attention on the early phase of B cell differentiation. *MS4A1*, which encodes CD20, a B lymphocyte surface molecule absent in plasma cells, was found overexpressed in this subcluster. No clear information emerged from H3a GenomicScape atlas screening. However, H3a GOEA underlined the following immune response processes: CXCR3 signaling pathway (*CXCL9*, *CXCL10*, *CXCL11*), TCR signaling pathway (*TRA*, *ICOS*, *ZAP70*, *MAP4K1*, *TARTP* [*CD3G*], *UBASH3A*), and T cell cytotoxicity (*GZMB*, *GSMH*, *GNLY*). To conclude, high expression of genes encoding for lymphorganogenic chemokines (*CCL19*, *CCL21*, *CXCL13*) and other TLS markers (*ICAM1*, *ICAM2*, *ICAM3*, *VCAM1*, *CCL22*) were observed in C3 versus C2 (*P* <  0.0001) [30, 31]. In brief, the presence of different B lymphocyte subsets in C3 suggested that complete B cell differentiation characterized this TNBC cluster and might be orchestrated with Th-1 cells and Tfh in TLS located in close proximity to tumors.

In TLS, intratumoral favorable immune phenotype relies on coordinated activation of pro-inflammatory genes, which include Th-1 transcripts (e.g., *IFNG*, *STAT1*, *IL12A*, *IL12B*, *IRF1*, *TBX21*, *CD8B*), Th-1 chemokine genes (e.g., *CXCL9*, *CXCL10*, *CCL5*), Tfh chemokine gene (*CXCL13*) and immune effector genes (e.g., *GNLY*, *GZMB*, *PRF1*), and B cells genes (e.g., *CD19*, *IGKC*) [29]. This immune process is accompanied by a counter activation of immunosuppressive mechanisms (e.g., *CD274* [*PD-L1*], *CTLA4*, *FOXP3*, *IDO1*, *PDCD1*), which will be discussed further [3, 32]. In our study, differential gene-expression analyses displayed a significant higher level of all except two genes (*FOXP3*, *CD8B*) in C3 compared to C2.

*CD274* (*PD-L1*), which is considered as an adaptive immune resistance marker in basal-like breast cancers, was overexpressed in C3 compared to C2 in internal and external cohorts (*P* <  0.0001) [33]. This information has to be pointed out because it may offer targeted therapeutic intervention for C3 patients.

#### Histological evaluation of TLS, TILs, B lymphocyte, and plasma cell stromal infiltrates confirmed transcriptomic results

Histological evaluation of TLS and lymphoid clusters showed that they were more frequent in C3 (10/12) than in C2 (9/22) (*P* = 0.0297) and in C1 (2/8) (*P* = 0.0194) (Fig. 3). No difference was found between C2 and C1. This result was corroborated by the presence of HEV (MECA79-positive) and follicular dendritic cell networks (CD21-positive) in C2 compared to C3: C2 (*n* = 22) vs C3 (*n* = 12), *P* <  0.0001 and *P* = 0.0017, respectively (Fig. 3). TILs displayed the following profile: C3 > C2 = C1 (*P* = 0.0014). Furthermore, plasma cell (CD138-positive cells) and B lymphocyte (CD20-positive cells) infiltrates were significantly higher in C3 compared to C2, *P* = 0.0297, and *P* = 0.0001, respectively (Fig. 3).

#### Immune response gradients between C2 and C3

To explore the possible existence of biological gradients between C2 and C3, we calculated correlation coefficients between C2 and C3 cluster probability orthogonal projection on C2-C3 axis and GES scores for each tumor (Additional file 5). Ten coefficients had absolute value superior to 0.5 (*P* <  0.0001) for internal cohort (Additional file 15). Nine of these correlations related to immune response GES. These results showed that anti-tumorigenic immune response decreases from C3 to C2 and, on the contrary, pro-tumorigenic immune response increases from C3 to C2.

#### Immune checkpoints screening

Expressions of 47 immune checkpoint genes were compared between C2 and C3 in internal and external cohorts [34]. Only significant and concordant results in both cohorts are discussed thereafter. Thirty-four immune checkpoints, including *CD274* (*PD-L1*), *CTLA4*, and *PDCD1* (*PD1*), displayed a C2 < C3 profile, five a C2 ≈ C3 profile, and one (*VTCN1*) a C2 > C3 profile (Additional file 16). Higher expression of a large majority (85%) of immune checkpoint genes was observed in C3 compared to C2, which was characterized by a marked immune response. This finding should be interpreted as a consequence of an immune response against tumor cells. In other words, immune inhibitory response by means of immune checkpoint upregulation could be triggered by a high anti-tumorigenic immune response to fine-tune and limit global immune response, i.e., to maintain immune self-tolerance. In regard to these results, we can hypothesize that immune checkpoints do not represent the main effectors of C2 immunosuppression and that they might contribute to the inefficacy of C3 anti-tumor immune attack.

#### External cohort annotation: fuzzy clustering followed by clinico-pathological, GOEA, and GES annotations

The same strategy as the one used for our internal cohort was applied on a TNBC external cohort to validate unsupervised analysis results based on the intersection of most variable probe sets in each cohort (*n* = 454). Internal and external TNBC subtyping results were then compared.

Fuzzy clustering separated external TNBC patients into three clusters, named C’1 (*n* = 61; 23.8%), C’2 (*n* = 97; 37.7%), and C’3 (*n* = 99; 38.5%). Naming of external TNBC clusters was based on biological correspondence with internal TNBC clusters (e.g., C’1 for C1). Distribution of patients among the three clusters was similar (Additional file 17).

C’1 TNBC patients were older than C’2 and C’3 TNBC patients (*P* = 0.0002). SBR1-2 histological grades were overrepresented in C’1 compared to C’2 and C’3 (*P* = 0.0134) (Additional file 18). Prognosis of TNBC patients belonging to C’3 showed no difference compared to C’2 patients (*P* = 0.10). No statistical difference was found for cluster and SBR distributions and age according to the internal and external cohort as a whole or split into the three clusters (Additional files 18 and 19).

All categorical GES distributions, except for PAM50, were quite similar (Fig. 1 and Additional files 9 and 20). The main difference regarding PAM50 was a high proportion of HER2E subtype in C’1 (47.5%) contrary to C1 (0%) and a low proportion of Luminal B subtype in C’1 (6.5%) contrary to C1 (54.5%). In fact, C1 is mostly composed of molecular apocrine subtypes, which are known to be characterized by luminal and HER2 features [18–21]. Furthermore, in C1, correlation coefficients with PAM50 Luminal B and HER2E centroids were close (data not shown). Here, subtype assignation should rather be “luminal B and HER2E,” i.e., molecular apocrine. Our conclusion is that PAM50 subtype assignment should be considered cautiously when used for TNBC subtyping, in contrast to CIT or TNBCtype GES which seem more appropriate.

Thirty-two out of 47 (68%) continuous score GES displayed exactly the same profile between internal and external cohort (Fig. 2 and Additional files 10 and 21). When selecting GES with two similarities, 40 out of 47 (85%) GES gave the same information.

CIBERSORT analysis showed that seven out of 22 immune cells were differentially distributed among C’2 and C’3 (Additional file 22). These profiles were the same as those observed in our internal cohort. C’2 tumors were infiltrated by three pro-tumorigenic immune cells and C’3 by four anti-tumorigenic immune cells.

When investigating the presence of an immune response gradient between C’2 and C’3, as was done on internal cohort, results corroborated what had been found: a continuous decrease of anti-tumorigenic immune response and continuous increase of pro-tumorigenic immune response from C’3 to C’2 (Additional file 23).

The same GOEA process was applied to the external cohort. Four clusters named H’1, H’2a, H’2b, and H’3 of highly expressed genes were visually individualized from heatmap composed of the 5% most variable probe sets. GOEA result comparison showed that the three clusters of the internal and external cohort were very similar (Additional files 12 and 13).

In conclusion, independent fuzzy clustering and functional annotation of the three clusters by means of clinicopathologic characteristics, GES, and GOEA indicated that the same molecular entities defined the different clusters in both the internal and external cohorts.

#### Non-TNBC external data testing

In regard to previous results, C1 can be considered as the less aggressive TNBC subtype. The question is now: What are the differences between C1 subtype and non-TNBC, from a biological point of view? In order to answer this question, non-TNBC external data were used.

PCA of TNBC (*n* = 257) and non-TNBC (*n* = 894) external data showed that C’1 patients were close to non-TNBC patients in the first plane (Additional file 24). PC1 separated non-TNBC and C’1 from basal-like clusters (C’2 and C’3) and PC2 separated C’2 and C’3 basal-like clusters as also observed for internal TNBC cohort. Interpretation of PCA result leads us to think that non-TNBC and non-basal-like TNBC probably share some biological similarities.

GES functional annotation comparison of C’1 (TNBC) and non-TNBC tumors showed both similarities and differences (Additional files 25, 26, 27 and 28). Among the main differences were intermediate prognostic scores for the three prognostic GES for C’1, highest for C’2 and C’3, and lowest for non-TNBC. Other results (ER, EGFR, and p53 GES scores) pointed out that aggressiveness of C’1 breast tumors was intermediate between non-TNBC and basal-like tumors. In conclusion, external data analysis showed that C’1 patients were closer to non-TNBC patients and that aggressiveness of C’1 tumors was intermediate between non-TNBC and basal-like tumors (C’2 and C’3).

## Discussion

The findings of this study strongly strengthen the fact that TNBC can be divided into three subtypes with potential therapeutic implications. To the best of our knowledge, we identified for the first time links between neurogenesis, tertiary lymphoid structures, plasma cells, B lymphocytes, and triple-negative breast cancer subtypes (C2 and C3).

Overall, our data show that IHC-typed TNBC regroup three different molecular subtypes of tumors, which would necessitate different and appropriate therapies. C1 is clearly a molecular apocrine cluster, which displays luminal, PIK3CA-mutated, and HER2E hallmarks. Use of non-TNBC data to refine C1 cluster annotation permitted to take into account relativity, which can weaken TNBC subtyping interpretation. When taking into account all breast cancers (non-TNBC and TNBC), C1 has to be considered as an intermediate group, from a biological aggressiveness point of view, between non-TNBC and basal-like enriched clusters.

C2 and C3 displayed basal-like hallmarks. However, these two basal-like enriched clusters showed a major biological discrepancy relative to immune response, which was characterized by a decreasing anti-tumorigenic immune gradient from C3 to C2 and a decreasing pro-tumorigenic gradient from C2 to C3. Furthermore, high neurogenesis activity was found for C2 tumors. In addition to immune response, the immune system is also known to play a pivotal role in tissue repair and regeneration. It is astonishing to notice that these two roles seem to be illustrated in C3 and C2, respectively.

We will not extensively develop the list of treatments, including immunotherapies, which could be proposed for TNBC patients, as recent review articles have been published on this topic [35]. We will select and discuss a few points, which concern the potential therapeutic ramifications of our findings and the treatment that might be used in function of cluster functional annotation (Additional file 29).

C1 tumors (molecular apocrine) could potentially be treated by antiandrogens or, better, by an association of PI3K inhibitors and antiandrogens, which demonstrated a synergistic anti-tumor effect [17]. Despite the fact that TNBC are defined by HER2 IHC negativity, *ERBB2* GES displayed high expression of *ERBB2* pathway in C1 tumors. The value of targeting the *ERBB2* pathway in C1 tumors warrants further investigations.

Immunotherapies, which aim to combat immunosuppression or stimulate adaptive immune response, could potentially be proposed for C2 (basal-like pro-tumorigenic immune response) and C3 (basal-like adaptive immune response) patients, respectively [36]. TLS evaluation and targeting represent a promising approach for the design of immune-based strategies which aimed at stimulating C3 patient immune response [37]. Therapeutic regimens should associate different immune checkpoint inhibitors, to enhance global efficacy and cytotoxicity of chemotherapy, because immunomodulators are not directly cytotoxic against tumor cells.

Immune checkpoints screening showed that upregulation of these markers characterized C3 compared to C2. We do not know if this immune response limitation is responsible for the global inefficacy of anti-tumor immune attack. Whatever the solution is, we can hypothesize that immune checkpoint inhibition should reinforce immune response against tumor cells in C3 [38]. However, *VTCN1* (*B7-H4*) displayed the only C2 > C3 profile. This gene codes for a B7 immunoregulatory protein, which exerts an immunosuppressive effect through inhibition of T cell activation, proliferation, and clonal expansion, and is considered as a protumorigenic factor [39]. In patients with ovarian carcinoma and glioma, macrophages expressing *VTCN1* have been directly linked to inhibition of T cell immune response [40, 41]. Therefore, *VTNC1* might actively participate in C2 immunosuppressive phenotype.

Tumor-associated macrophages are crucial actors of tumor fate and therefore represent important and promising immunotherapeutic targets [42–45]. Consequently, numerous macrophage-directed therapeutic approaches are under investigation and should take into account M2/M1 macrophage status according to TNBC subtype.

C2 patients could potentially be considered for anti-neurogenic therapies as recent studies have unraveled the role of neurogenesis in cancer progression and anti-neurogenic therapies are emerging in oncology [46–48]. In prostate, gastric, skin and pancreatic cancers, it has been shown that the infiltration of new nerves in the tumor microenvironment is necessary to primary tumor growth and metastasis [49–52]. The release of neurotransmitters by nerve endings results in the stimulation of corresponding receptors in both stromal and cancer cells, leading to increased tumor growth and dissemination. In particular, the release of noradrenaline by sympathetic nerves induces an angiogenic switch via the stimulation of beta adrenergic receptors in endothelial cells [53]. In breast cancer, neurogenesis has been reported in the tumor microenvironment, in particular from nerves of sympathetic origin, and the density of nerve infiltration is associated with cancer aggressiveness [54]. Neurogenesis was found across all breast cancer subtypes, including TNBC. Our present study shows that neurogenesis-related gene expression level is more specifically increased in the C2 cluster of TNBC, suggesting that anti-neurogenic therapies, such as those targeting the neurotrophic tyrosine kinase receptor 1 (NTRK1) could be relevant in C2 TNBC [46].

## Conclusions

Three TNBC subtypes were characterized in this work: one molecular apocrine and two basal-like with, inter alia, opposite immune responses. Some of these marked biological features could be targeted by specific therapies. Various targeted therapies could be tested in combination to produce synergistic effects and prevent resistance in the different subtypes of TNBC identified here. Today, molecular heterogeneity of TNBC participates in the limited efficacy of therapies used in unsubtyped TNBC patients. There is a clear need to test, or re-test, new or old targeted therapies, in new clinical trials taking into account these three TNBC subtypes.

## Additional files

Additional file 1: External TNBC and non-TNBC genomic data. (PDF 87 kb)

Additional file 2: GES list, characteristics, bioinformatics, and references. (PDF 197 kb)

Additional file 3: Details of antibodies used for immunohistochemistry. (PDF 84 kb)

Additional file 4: Projection of internal TNBC cohorts in the first PCA plane. **(A)** PACS08 patients (*n* = 131, [orange]) compared to TNBC patients of a previous study (*n* = 107, [black]). **(B)** Each color represents a recruitment site, except for grayish pale green color, which represents one or two patients recruited in different sites (*n* = 36 patients; 28 sites). Total number of recruitment sites was equal to 45. (PDF 95 kb)

Additional file 5: Fuzzy clustering of 238 TNBC. Distribution of patients based on probability of belonging to cluster: C1, *n* = 55 (blue); C2, *n* = 98 (red) and C3, *n* = 85 (green). Each vertex of the triangle represents a cluster and each point represents a patient, placed as the barycenter of the triangle, weights being the probabilities of belonging to each of the clusters. The closer a point is to one of the vertices, the greater is the probability of the patient to belong to the corresponding cluster. (PDF 86 kb)

Additional file 6: Event-free survival analysis of TNBC pooled cohort. This cohort is composed of 427 patients from internal (*n* = 238) and external (*n* = 189) cohorts (C1 & C’1 [blue]; C2 & C’2 [red] and C3 & C’3 [green]). (PDF 141 kb)

Additional file 7: Projection of internal TNBC cohort in the first PCA plane. (C1, *n* = 55 [blue]; C2, *n* = 98 [red]; C3, *n* = 85 [green]). (PDF 88 kb)

Additional file 8: PCA of internal TNBC cohort. Lists of PC1 and PC2 twenty highest absolute weight probes. (PDF 115 kb)

Additional file 9: Categorical GES analyses result in function of internal TNBC clusters (C1, C2, and C3). (PDF 107 kb)

Additional file 10: Continuous score GES analyses result in function of internal TNBC clusters (C1, C2, and C3). (PDF 176 kb)

Additional file 11: Significant EFS analyses based on GES scores. **(A)** Internal and **(B)** external C2 and C3 TNBC patients. (PDF 103 kb)

Additional file 12: ToppGene GOEA based on heatmap gene clusters, internal cohort (1843 probes) and external cohorts (454 probes). Identical GO terms per cluster of highly expressed genes are indicated in bold characters. (PDF 143 kb)

Additional file 13: ToppGene GOEA based on SAM gene lists for the three clusters, internal and external cohorts. Identical GO terms per SAM lists are indicated in bold characters. (PDF 94 kb)

Additional file 14: B lymphocyte lineage GenomicScape analysis in function of the three H3 subclusters (H3a, H3b, H3c). Genes upregulated in the given group compared to the overall mean expression are highlighted in red. Genes downregulated in the given group compared to the overall mean expression are highlighted in blue. (PDF 149 kb)

Additional file 15: Biological gradients between C2 and C3 in our internal cohort. Correlation coefficient calculation between GES score for each internal cohort tumor (x number), and C2 and C3 cluster probability orthogonal projection on C2-C3 axis (y number) (C2 tumor, red; C3 tumor, green). Only correlation coefficient absolute values superior to 0.5 (*P* <  0.0001) are displayed. (PDF 101 kb)

Additional file 16: Expression of immune checkpoints between basal-like clusters. Internal (C2 vs C3) and external (C’2 vs C’3) cohorts. (PDF 158 kb)

Additional file 17: Comparison of internal and external TNBC cohorts’ clinicopathologic characteristics. (PDF 135 kb)

Additional file 18: External TNBC cohort’s clinicopathologic characteristics in function of the three clusters (C’1, C’2, and C’3). (PDF 136 kb)

Additional file 19: Comparison of clinicopathologic characteristics of TNBC cohorts in function of clusters. Internal (C1, *n* = 55; C2, *n* = 98; C3, *n* = 85) and external (C’1, *n* = 61; C’2, *n* = 97; C’3, *n* = 99). (PDF 138 kb)

Additional file 20: Categorical GES analyses result in function of external TNBC clusters (C’1, C’2 and C’3). (PDF 159 kb)

Additional file 21: Continuous score GES analyses results in function of external TNBC clusters (C’1, C’2, and C’3). (PDF 228 kb)

Additional file 22: C’2 vs C’3 CIBERSORT analysis results. (PDF 199 kb)

Additional file 23: Biological gradients between C2 and C3 in an external cohort. Correlation coefficient calculation between GES score for each external cohort tumor (x number), and C’2 and C’3 cluster probability orthogonal projection on C’2-C’3 axis (y number) (C’2 tumor, red; C’3 tumor, green). Only correlation coefficient absolute values superior to 0.5 (*P* <  0.0001) are displayed. (PDF 155 kb)

Additional file 24: PCA result representation of external TNBC and non-TNBC data. PCA is computed based on the TNBC data and the values of the two first principal components (PC1 and PC2) are then predicted for the non-TNBC data. All patients are then projected onto the plane generated by PC1 and PC2. Ellipses represent the 95% confidence of belonging to the clusters data (C’1, *n* = 61 [blue]; C’2, *n* = 97 [red] and C’3, *n* = 99 [green]). External non-TNBC data (*n* = 894) are figured by black points. (PDF 149 kb)

Additional file 25: Categorical GES analyses interpretation in function of external TNBC (C’1, C’2, C’3) and non-TNBC clusters (NTN). (PDF 238 kb)

Additional file 26: Continuous score GES analyses results in function of external TNBC (C’1, C’2, C’3) and non-TNBC clusters (NTN). (PDF 245 kb)

Additional file 27: Categorical GES distributions in function of external TNBC (C’1, C’2, C’3) and non-TNBC clusters (NTN). (A) CIT, (B) ER-negative and (C) PAM50. (PDF 138 kb)

Additional file 28: Molecular annotation of external cohort in function of TNBC clusters and non-TNBC. Radar plot showing the segregation of three clusters as a function of 47 continuous score GES. This figure is an illustration of Additional file 26. Each of the 47 radii represents a GES. Black circles represent significantly different levels of expression from low (smallest circle) to high (largest circle). Expression level of each cluster is represented on the corresponding circle as a dark gray (NTN), blue (C’1), red (C’2) or green (C’3) dot. Dots located on same circles correspond to clusters with not significantly different expressions. Dots located on different circles correspond to clusters with significantly different expressions. Dots located in between black circles correspond to a cluster with expression level not significantly different from clusters whose dots are located on both near circles. (PDF 287 kb)

Additional file 29: Proposed therapeutic strategies in function of GES and robust subtyping results. (PDF 162 kb)

## Abbreviations

ANOVA: One-way analysis of variance
AR: Androgen receptor
CIBERSORT: Cell type Identification By Estimating Relative Subsets Of known RNA Transcripts
EFS: Event-free survival
ER: Estrogen receptor
GEO: Gene expression omnibus
GES: Gene expression signature
GO: Gene ontology
GOEA: Gene ontology enrichment analysis
HER2: Epidermal growth receptor 2
HEV: High endothelial venules
IHC: Immunohistochemistry
PCA: Principal component analysis
PNI: Perineural invasion
PR: Progesterone receptor
SAM: Significance analysis of microarray
TIL: Tumor infiltrating lymphocyte
TLS: Tertiary lymphoid structure
TMA: Tissue microarrays
TNBC: Triple-negative breast cancer
