# Supplementary material for: Identification of three subtypes of triple-negative breast cancer with potential therapeutic implications
Source: Breast Cancer Res. 2019 May 17;21:65. doi: 10.1186/s13058-019-1148-6 (PMC6525459; doi:10.1186/s13058-019-1148-6)
Supplement: Supplementary file 1 — External TNBC and non-TNBC genomic data. (PDF 87 kb) [file 13058_2019_1148_MOESM1_ESM.pdf]

## Additional file 1: External TNBC and non-TNBC genomic data.

| Study code | DNA chip | TNBC patients | Non-TNBC patients | Reference |
|---|---|---|---|---|
| GSE12276 | Affymetrix U133P2 | 56 | 148 | (S35) |
| GSE18864 | Affymetrix U133P2 | 35 | 40 | (S36) |
| GSE19615 | Affymetrix U133P2 | 28 | 87 | (S37) |
| GSE21653 | Affymetrix U133P2 | 87 | 170 | (S38) |
| E-MTAB-365 | Affymetrix U133P2 | 51 | 449 | (S39) |
| | | **257** | **894** | |

## References

S35. Bos PD, Zhang XH, Nadal C, Shu W, Gomis RR, Nguyen DX et al. Genes that mediate breast cancer metastasis to the brain. Nature. 2009;459:1005-9.

S36. Silver DP, Richardson AL, Eklund AC, Wang ZC, Szallasi Z, Li Q et al. Efficacy of neoadjuvant Cisplatin in triple-negative breast cancer. J Clin Oncol. 2010;28:1145-53.

S37. Li Y, Zou L, Li Q, Haibe-Kains B, Tian R, Li Y et al. Amplification of LAPTM4B and YWHAZ contributes to chemotherapy resistance and recurrence of breast cancer. Nat Med. 2010;16:214-8.

S38. Sabatier R, Finetti P, Cervera N, Lambaudie E, Esterni B, Mamessier E et al. A gene expression signature identifies two prognostic subgroups of basal breast cancer. Breast Cancer Res Treat. 2011;126:407-20.

S39. Guedj M, Marisa L, de Reynies A, Orsetti B, Schiappa R, Bibeau F et al. A refined molecular taxonomy of breast cancer. Oncogene. 2011;31:1196-206.
