# Supplementary material for: Identification of three subtypes of triple-negative breast cancer with potential therapeutic implications
Source: Breast Cancer Res. 2019 May 17;21:65. doi: 10.1186/s13058-019-1148-6 (PMC6525459; doi:10.1186/s13058-019-1148-6)
Supplement: Supplementary file 2 — GES list, characteristics, bioinformatics, and references. (PDF 197 kb) [file 13058_2019_1148_MOESM2_ESM.pdf]

Additional file 2: GES list, characteristics, bioinformatics and references.

N°	GES name	*n* genes	*n* probes	Statistics	References
**Molecular subtyping**					
1	4-TNBC	80	-	Nearest centroid classifier	(S1)
2	AR	36	-	Weighted average expression	(S2)
3	Basal-like	-	37	Average expression	(S3)
4	CIT	-	375	Nearest centroid classifier	(S4)
5	Claudin-CD24	-	19	Average expression	(S3)
6	Claudin-low	807	-	Nearest centroid classifier	(S5)
7	ER	-	135	Weighted average expression	(S6)
8	ER-negative	813	-	Nearest centroid classifier	(S7)
9	ERBB2	-	4	Weighted average expression	(S6)
10	Molecular-apocrine	-	27	Average expression	(S3)
11	PAM50	50	-	Nearest centroid classifier	(S8)
12	TNBCtype	2188	-	Nearest centroid classifier	(S9)
**Immune response**					
13	B-cell	-	48	Average expression	(S3)
14	CIBERSORT	547	-	v-support vector regression with linear kernel	(S10)
15	CYT	-	2	Average expression	(S11)
16	IL-8	-	4	Average expression	(S3)
17	M2/M1	649	-	Weighted average expression	(S12)
18	M2/M1 (Becker)	164	-	Weighted average expression	(S13)
19	MHC-1	-	17	Average expression	(S3)
20	MHC-2	-	14	Average expression	(S3)
21	STAT1	-	14	Average expression	(S14)
22	T-cell	-	27	Average expression	(S3)
23	Type I IFN	-	14	Average expression	(S3)
**Microenvironment cells**					
24	Epithelial cells	207	-	Single-sample gene set enrichment analysis (ssGSEA)	(S15)
25	Fibroblasts	189	-	Single-sample gene set enrichment analysis (ssGSEA)	(S15)
26	Neurons	219	-	Single-sample gene set enrichment analysis (ssGSEA)	(S15)
**Metabolisms**					
27	Adipocytes	-	8	Average expression	(S3)
28	Glycolysis	6	-	Average expression	(S16)
29	IRGS	-	19	Weighted average expression	(S17)
**Critical biological pathways in cancer**					
30	AKT	379	-	Weighted average expression	(S18)
31	β-Catenin	-	98	Weighted average expression	(S19)
32	CIN	25	-	Average expression	(S20)
33	E2F3	-	298	Weighted average expression	(S19)
34	EGFR	27	-	Average expression	(S21)
35	HOXA	-	8	Average expression	(S3)
36	MITO/OXPHOS	38	-	average expression	(S22)
37	MYC	-	248	Weighted average expression	(S19)
38	p53	-	32	Weighted average expression	(S23)
39	PIK3CA	-	278	Weighted average expression	(S24)
40	PNI	-	24	Weighted average expression	(S25)
41	PRL	75	-	Average expression	(S26)
42	Proliferation	-	47	Average expression	(S3)
43	PTEN loss	246	-	Weighted average expression	(S27)
44	RAS	-	348	Weighted average expression	(S19)
45	Reactive stroma	-	47	Average expression	(S3)
46	SRC	-	73	Weighted average expression	(S19)
47	Stroma-CD10	12	-	Weighted average expression	(S28)
48	TGFβ	-	55	Weighted average expression	(S29)
49	VEGF	-	7	Average expression	(S3)
50	Wound-response	459	-	Weighted average expression	(S30)
51	YAP1-WWTR1	24	-	Average expression	(S31)
**Prognosis**					
52	38-GES	38	-	Weighted average expression	(S32)
53	70-GES	70	-	Nearest centroid classifier	(S33)
54	GGI	-	128	Weighted average expression	(S34)

References

S1. Burstein MD, Tsimelzon A, Poage GM, Covington KR, Contreras A, Fuqua SA et al. Comprehensive genomic analysis identifies novel subtypes and targets of triple-negative breast cancer. Clin Cancer Res. 2015;21:1688-98.

S2. Naderi A. Coagulation factor VII is regulated by androgen receptor in breast cancer. Exp Cell Res. 2015; 331:239-50.

S3. Karn T, Pusztai L, Holtrich U, Iwamoto T, Shiang CY, Schmidt M et al. Homogeneous datasets of triple negative breast cancers enable the identification of novel prognostic and predictive signatures. PLoS One. 2011;6:e28403.

S4. Guedj M, Marisa L, de Reynies A, Orsetti B, Schiappa R, Bibeau F et al. A refined molecular taxonomy of breast cancer. Oncogene. 2012;31:1196-206.

S5. Prat A, Parker JS, Karginova O, Fan C, Livasy C, Herschkowitz JI et al. Phenotypic and molecular characterization of the claudin-low intrinsic subtype of breast cancer. Breast Cancer Res. 2010;12:R68.

S6. Wolf DM, Lenburg ME, Yau C, Boudreau A, van't Veer LJ. Gene co-expression modules as clinically relevant hallmarks of breast cancer diversity. PLoS One. 2014;9:e88309.

S7. Teschendorff AE, Miremadi A, Pinder SE, Ellis IO, Caldas C. An immune response module identifies a good prognosis subtype in estrogen negative breast cancer. Genome Biol. 2007;8:R157.

S8. Sorlie T, Tibshirani R, Parker J, Hasties T, Marron JS, Nobel A et al. Repeated observation of breast tumor subtypes in independent gene expression data sets. Proc Natl Acad Sci USA. 2003;100:8418-23.

S9. Lehmann BD, Bauer JA, Chen X, Sanders ME, Chakravarthy AB, Shyr Y et al. Identification of human triple-negative breast cancer subtypes and preclinical models for selection of targeted therapies. J Clin Invest. 2011;121:2750-67.

S10. Newman AM, Liu CL, Green MR, Gentles AJ, Feng W, Xu Y et al. Robust enumeration of cell subsets from tissue expression profiles. Nat Methods. 2015;12:453-57.

S11. Rooney M, Shukla SA, Wu CJ, Getz G, Hacohen N: Molecular and genetic properties of tumors associated with local immune cytolytic activity. Cell. 2015;160:48-61.

S12. Jézéquel P, Loussouarn D, Guérin-Charbonnel C, Campion L, Vanier A, Gouraud W et al. Gene-expression molecular subtyping of triple-negative breast cancer tumours: importance of immune response. Breast Cancer Res. 2015;17:43.

S13. Becker M, De Bastiani MA, Parisi MM, Guma FT, Markoski MM, Castro MA et al. Integrated Transcriptomics Establish Macrophage Polarization Signatures and have Potential Applications for Clinical Health and Disease. Sci Rep. 2015;5:13351.

S14. Rody A, Holtrich U, Pusztai L, Liedtke C, Gaetje R, Ruckhaeberle E et al. T-cell metagene predicts a favorable prognosis in estrogen receptor-negative and HER2-positive breast cancers. Breast Cancer Res. 2009;11:R15.

S15. Aran D, Hu Z, Butte AJ. xCell: Digitally portraying the tissue cellular heterogeneity landscape. Genome Biol. 2017;18:220.

S16. Hu Z, Fan C, Livasy C, He X, Oh DS, Ewend MG et al. A compact VEGF signature associated with distant metastases and poor outcomes. BMC Med. 2009;7:9.

S17. Miller LD, Coffman LG, Chou JW, Black MA, Bergh J, D'Agostino R Jr et al. An iron regulatory gene signature predicts outcome in breast cancer. Cancer Res. 2011;71:6728-37.

S18. Majumder PK, Febbo PG, Bikoff R, Berger R, Xue Q, McMahon LM et al. mTOR inhibition reverses Akt-dependent prostate intraepithelial neoplasia through regulation of apoptotic and HIF-1-dependent pathways. Nat Med. 2004;10:594-601.

S19. Bild AH, Yao G, Chang JT, Wang Q, Potti A, Chasse D et al. Oncogenic pathway signatures in human cancers as a guide to targeted therapies. Nature. 2006;439:353-7.

S20. Carter SL, Eklund AC, Kohane IS, Harris LN, Szallasi Z. A signature of chromosomal instability inferred from gene expression profiles predicts clinical outcome in multiple human cancers. Nat Genet. 2006;9:1043-8.

S21. Hoadley KA, Weigman VJ, Fan C, Sawyer LR, He X, Troester MA et al. EGFR associated expression profiles vary with breast tumor subtype. BMC Genomics. 2007;8:258.

S22. Whitaker-Menezes D, Martinez-Outschoorn UE, Flomenberg N, Birbe RC, Witkiewicz AK, Howell A et al. Hyperactivation of oxidative mitochondrial metabolism in epithelial cancer cells in situ: visualizing the therapeutic effects of metformin in tumor tissue. Cell Cycle. 2011;23:4047-64.

S23. Miller LD, Smeds J, George J, Vega VB, Vergara L, Ploner A et al. An expression signature for p53 status in human breast cancer predicts mutations status, transcriptional effects, and patients survival. Proc Natl Acad Sci USA. 2005;102:13550–5.

S24. Loi S, Haibe-Kains B, Majjaj S, Lallemand F, Durbecq V, Larsimont D et al. PIK3CA mutations associated with gene signature of low mTORC1 signaling and better outcomes in estrogen receptor-positive breast cancer. Proc Natl Acad Sci. USA. 2010;107:10208-13.

S25. Mays AC, Chou J, Craddock AL, Miller L, Browne JD. Gene Variability Between Perineural-positive and Perineural-negative Squamous Cell Skin Cancers. Anticancer Res. 2016;36:4007-11.

S26. Sato T, Tran TH, Peck AR, Liu C, Ertel A, Lin J et al. Global profiling of prolactin-modulated transcripts in breast cancer in vivo. Mol Cancer. 2013;12:59.

S27. Saal LH, Johansson P, Holm K, Gruvberger-Saal SK, She QB, Maurer M et al. Poor prognosis in carcinoma is associated with a gene expression signature of aberrant PTEN tumor suppressor pathway activity. Proc Natl Acad Sci USA. 2007;104:7564-9.

S28. Desmedt C, Majjaj S, Kheddoumi N, Singhal SK, Haibe-Kains B, El Ouriaghli F et al. Characterization and clinical evaluation of CD10+ stroma cells in the breast cancer microenvironment. Clin Cancer Res. 2012;18:1004–14.

S29. Padua D, Zhang XH, Wang Q, Nadal C, Gerald WL, Gomis RR et al. TGFbeta primes breast tumors for lung metastasis seeding through angiopoietin-like 4. Cell. 2008;133: 66-77.

S30. Chang HY, Sneddon JB, Alizadeh AA, Sood R, West RB, Montgomery K et al. Gene expression signature of fibroblast serum response predicts human cancer progression: similarities between tumors and wounds. PLoS Biol. 2004;2:E7.

S31. Subramanian A, Tamayo P, Mootha VK, Mukherjee S, Ebert BL, Gillette MA et al. Gene set enrichment analysis: A knowledge-based approach for interpreting genome-wide expression profiles. Proc Natl Acad Sci USA. 2005;102:15545-50.

S32. Jézéquel P, Campone M, Roché H, Gouraud W, Charbonnel C, Ricolleau G et al. A 38-gene expression signature to predict metastasis risk in node-positive cancer after systemic adjuvant chemotherapy: a genomic substudy of PACS01 clinical trial. Breast Cancer Res Treat. 2009;116:509-20.

S33. van 't Veer LJ, Dai H, van de Vijver MJ, He YD, Hart AA, Mao M et al. Gene expression profiling predicts clinical outcome of breast cancer. Nature. 2002;415:530-6.

S34. Sotiriou C, Wirapati P, Loi S, Harris A, Fox S, Smeds J et al. Gene expression profiling in breast cancer: understanding the molecular basis of histologic grade to improve prognosis. J Natl Cancer Inst. 2006;98:262–72.
