# Supplementary material for: Identification of three subtypes of triple-negative breast cancer with potential therapeutic implications
Source: Breast Cancer Res. 2019 May 17;21:65. doi: 10.1186/s13058-019-1148-6 (PMC6525459; doi:10.1186/s13058-019-1148-6)
Supplement: Supplementary file 3 — Details of antibodies used for immunohistochemistry. (PDF 84 kb) [file 13058_2019_1148_MOESM3_ESM.pdf]

**Additional file 3: Details of antibodies used for immunohistochemistry.**

| Target | Clone | Dilution | Antigen retrieval | Supplier |
|---|---|---|---|---|
| CD20 | L26 | Prediluted | CC1 Ventana (pH 8.4) | Ventana |
| CD21 | 2G9 | Prediluted | CC1 Ventana (pH 8.4) | Ventana |
| CD138/syndecan-1 | B-A38 | Prediluted | CC1 Ventana (pH 8.4) | Ventana |
| MECA79 | MECA79 | 1/200 | CC1 Ventana (pH 8.4) | Santa Cruz |
| UCHL1/PGP9.5 | Polyclonal | 1/400 | CC1 Ventana (pH 8.4) | Abcam |
| S100 | Polyclonal | Prediluted | None | Ventana |
