# Supplementary material for: Identification of three subtypes of triple-negative breast cancer with potential therapeutic implications
Source: Breast Cancer Res. 2019 May 17;21:65. doi: 10.1186/s13058-019-1148-6 (PMC6525459; doi:10.1186/s13058-019-1148-6)

Additional file 4: Projection of internal TNBC cohorts in the first PCA plane. (A) PACS08 patients (n = 131, [orange]) compared to TNBC patients of a previous study (n = 107, [black]). **(B)** Each color represents a recruitment site, except for greyish pale green color, which represents one or two patients recruited in different sites (n = 36 patients; 28 sites). Total number of recruitment sites was equal to 45.

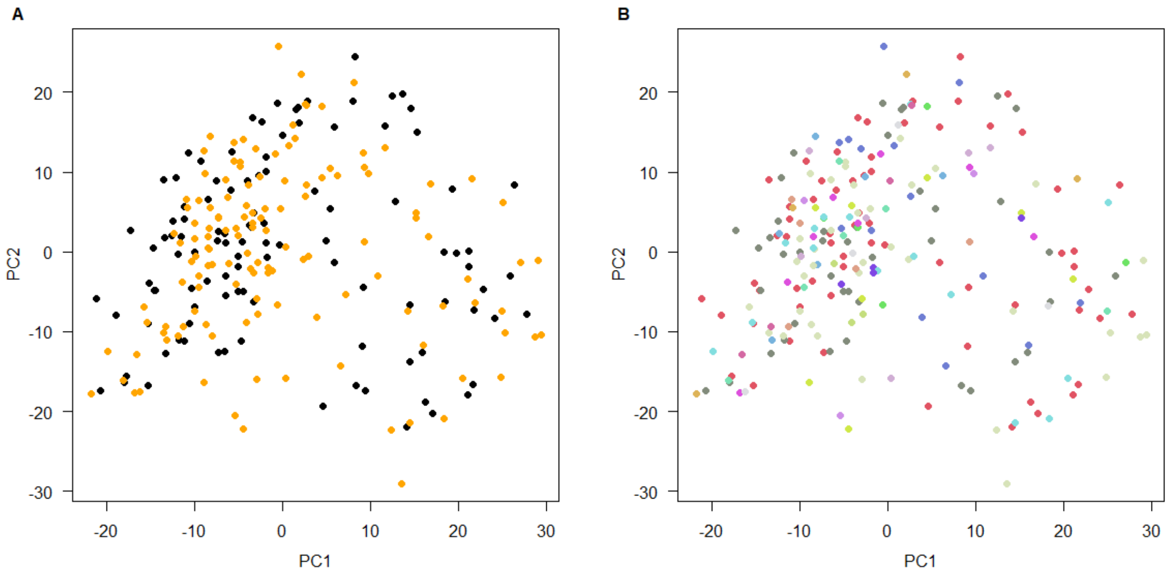

Supplement: Supplementary file 4 — Projection of internal TNBC cohorts in the first PCA plane. (A) PACS08 patients (n = 131, [orange]) compared to TNBC patients of a previous study (n = 107, [black]). (B) Each color represents a recruitment site, except for grayish pale green color, which represents one or two patients recruited in different sites (n = 36 patients; 28 sites). Total number of recruitment sites was equal to 45. (PDF 95 kb) [file 13058_2019_1148_MOESM4_ESM.pdf]
