# Supplementary material for: Identification of three subtypes of triple-negative breast cancer with potential therapeutic implications
Source: Breast Cancer Res. 2019 May 17;21:65. doi: 10.1186/s13058-019-1148-6 (PMC6525459; doi:10.1186/s13058-019-1148-6)

Additional file 5: Fuzzy clustering of 238 TNBC. Distribution of patients based on probability of belonging to cluster: C1, n = 55 (blue); C2, n = 98 (red) and C3, n = 85 (green). Each vertex of the triangle represents a cluster and each point represents a patient, placed as the barycenter of the triangle, weights being the probabilities of belonging to each of the clusters. The closer a point is to one of the vertices, the greater is the probability of the patient to belong to the corresponding cluster.

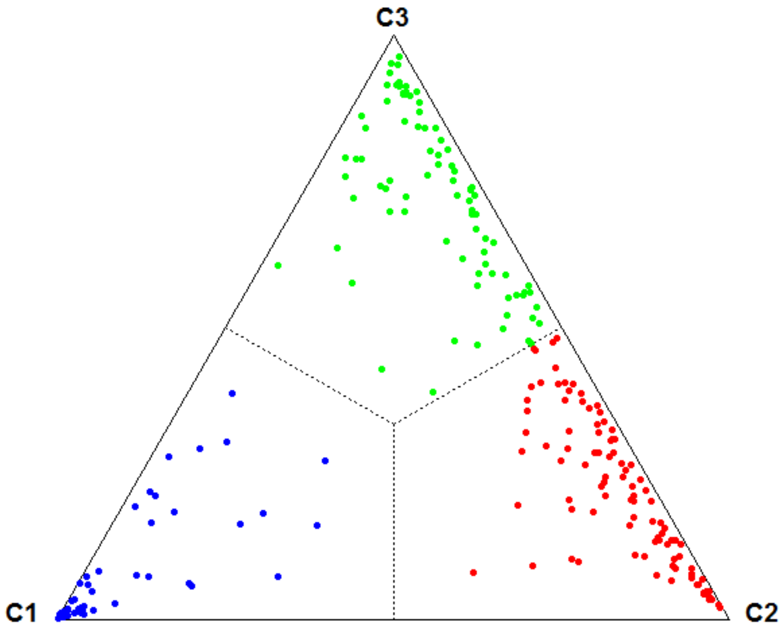

Supplement: Supplementary file 5 — Fuzzy clustering of 238 TNBC. Distribution of patients based on probability of belonging to cluster: C1, n = 55 (blue); C2, n = 98 (red) and C3, n = 85 (green). Each vertex of the triangle represents a cluster and each point represents a patient, placed as the barycenter of the triangle, weights being the probabilities of belonging to each of the clusters. The closer a point is to one of the vertices, the greater is the probability of the patient to belong to the corresponding cluster. (PDF 86 kb) [file 13058_2019_1148_MOESM5_ESM.pdf]
