# Supplementary material for: Identification of three subtypes of triple-negative breast cancer with potential therapeutic implications
Source: Breast Cancer Res. 2019 May 17;21:65. doi: 10.1186/s13058-019-1148-6 (PMC6525459; doi:10.1186/s13058-019-1148-6)

Additional file 6: Event-free survival analysis of TNBC pooled cohort. This cohort is composed of 427 patients from internal (n = 238) and external (n = 189) cohorts (C1 & C’1 [blue]; C2 & C’2 [red] and C3 & C’3 [green]).

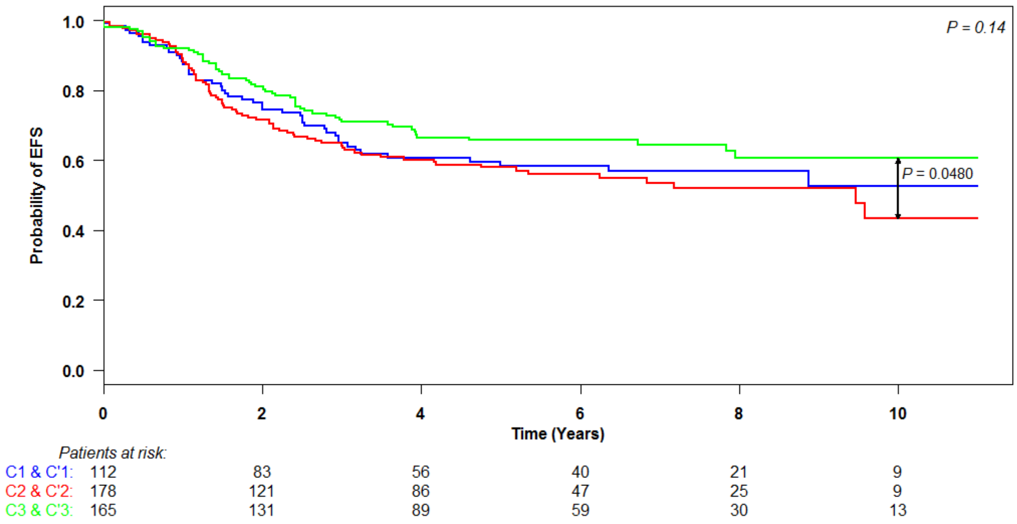

Supplement: Supplementary file 6 — Event-free survival analysis of TNBC pooled cohort. This cohort is composed of 427 patients from internal (n = 238) and external (n = 189) cohorts (C1 & C’1 [blue]; C2 & C’2 [red] and C3 & C’3 [green]). (PDF 141 kb) [file 13058_2019_1148_MOESM6_ESM.pdf]
