# Supplementary material for: Identification of three subtypes of triple-negative breast cancer with potential therapeutic implications
Source: Breast Cancer Res. 2019 May 17;21:65. doi: 10.1186/s13058-019-1148-6 (PMC6525459; doi:10.1186/s13058-019-1148-6)

Additional file 7: Projection of internal TNBC cohort in the first PCA plane. (C1, *n* = 55 [blue]; C2, *n* = 98 [red]; C3, *n* = 85 [green]).

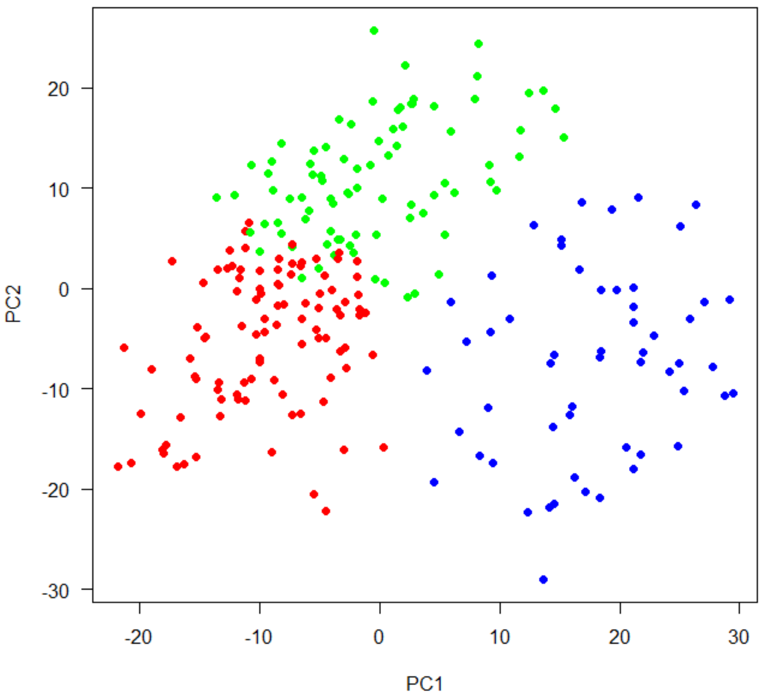

Supplement: Supplementary file 7 — Projection of internal TNBC cohort in the first PCA plane. (C1, n = 55 [blue]; C2, n = 98 [red]; C3, n = 85 [green]). (PDF 88 kb) [file 13058_2019_1148_MOESM7_ESM.pdf]
