# Supplementary material for: Identification of three subtypes of triple-negative breast cancer with potential therapeutic implications
Source: Breast Cancer Res. 2019 May 17;21:65. doi: 10.1186/s13058-019-1148-6 (PMC6525459; doi:10.1186/s13058-019-1148-6)
Supplement: Supplementary file 8 — PCA of internal TNBC cohort. Lists of PC1 and PC2 twenty highest absolute weight probes. (PDF 115 kb) [file 13058_2019_1148_MOESM8_ESM.pdf]

Additional file 8: PCA of internal TNBC cohort. Lists of PC1 and PC2 twenty highest absolute weight probes.

PC1

**N°**	**Affymetrix probe set ID**	**Gene symbol**	**Weight**	**Biological pathway**	**Reference**
1	206509_at	*PIP*	0.0644	MA	(S40)
2	226192_at	*AR*	0.0635	MA	(S40)
3	237086_at	*FOXA1*	0.0629	MA	(S40)
4	1553394_a_at	*TFAP2B*	0.0621	MA, PIK3CAm	(S40, S41)
5	1553434_at	*CYP4Z2P*	0.0617	PIK3CAm	(S41)
6	237339_at	*LINC00993*	0.0612	PIK3CAm	(S42)
7	237395_at	*CYP4Z1*	0.0609	PIK3CAm	(S41)
8	223864_at	*ANKRD30A*	0.0601	PIK3CAm	(S41)
9	232105_at	*BLACAT1*	-0.0600		
10	214451_at	*TFAP2B*	0.0596	MA, PIK3CAm	(S40, S41)
11	204667_at	*FOXA1*	0.0595	MA	(S40)
12	206165_s_at	*CLCA2*	0.0588	MA	(S40)
13	228969_at	*AGR2*	0.0585	MA	(S43)
14	204607_at	*HMGCS2*	0.0580	MA, PIK3CAm	(S40, S41)
15	222904_s_at	*TMC5*	0.0578	PIK3CAm	(S41)
16	220192_x_at	*SPDEF*	0.0577	MA	(S40)
17	217528_at	*CLCA2*	0.0573	MA	(S40)
18	206164_at	*CLCA2*	0.0571	MA	(S40)
19	1556474_a_at	*FLJ38379*	0.0569		
20	219580_s_at	*TMC5*	0.0566	PIK3CAm	(S41)

PC2

**N°**	**Affymetrix probe set ID**	**Gene symbol**	**Weight**	**Biological pathway**	
1	205267_at	*LOC101928620 /// POU2AF1*	0.0802		
2	217480_x_at	*IGKV1OR1-1 /// IGKV1OR10-1 /// IGKV1OR2-1 /// IGKV1OR2-118*	0.0797		
3	217179_x_at	No gene	0.0797		
4	211798_x_at	*IGLJ3*	0.0795	B cell LD	(S44)
5	234764_x_at	*CKAP2 /// DKFZp667J0810 /// IGLC1 /// IGLJ2 /// IGLJ2 /// IGLJ3 /// IGLJ3 /// IGLJ3 /// IGLV1-36 /// IGLV1-36 /// IGLV1-44 /// IGLV1-50 /// IGLV1-50 /// IGLV@*	0.0795		
6	217227_x_at	*IGLV1-44*	0.0793	B cell LD	(S44)
7	214916_x_at	*IGHA1 /// IGHA2 /// IGHG1 /// IGHG3 /// IGHJ4 /// IGHM /// IGHV3-23 /// IGHV4-31 /// IGK /// ZCWPW2*	0.0791		
8	216510_x_at	*IGHA1 /// IGHG1 /// IGHM /// IGHV3-23 /// IGHV4-31 /// IGK /// ZCWPW2*	0.0790		
9	236191_at	No gene	0.0790		
10	217157_x_at	No gene	0.0784		
11	211650_x_at	*IGH /// IGHA1 /// IGHD /// IGHG1 /// IGHG3 /// IGHM /// IGHV3-23 /// IGHV4-31*	0.0783		
12	211881_x_at	*IGLJ3*	0.0783	B cell LD	(S44)
13	214768_x_at	*IGKV2-28 /// IGKV2D-28*	0.0782		
14	216557_x_at	*IGHA1 /// IGHD /// IGHG1 /// IGHG3 /// IGHM /// IGHV3-21 /// IGHV3-48 /// IGHV3-71 /// IGHV4-31*	0.0781		
15	215946_x_at	*IGLL3P*	0.0780	B cell LD	(S44)
16	216207_x_at	*IGKC*	0.0780	B cell LD	(S44)
17	211645_x_at	*IGKV1-17*	0.0778	B cell LD	(S44)
18	217148_x_at	*IGLC1 /// IGLJ3 /// IGLV2-14 /// IGLV@*	0.0778		
19	217281_x_at	No gene	0.0777		
20	216401_x_at	*IGKV1-37 /// IGKV1D-37*	0.0775		

MA: molecular apocrine; PIK3CAm: genes up-regulated in PIK3CA-mutated ERalpha-positive breast cancer cells; B cell LD: B cell lineage differentiation

References

S40. Farmer P, Bonnefoi H, Becette V, Tubiana-Hulin M, Fumoleau P, Larsimont D et al. Identification of molecular apocrine breast tumours by microarray analysis. Oncogene. 2005;24:4660-71.

S41. Cizkova M, Cizeron-Clairac G, Vacher S, Susini A, Andrieu C, Lidereau R et al. Gene expression profiling reveals new aspects of PIK3CA mutation in ERalpha-positive breast cancer: major implication of the Wnt signaling pathway. PLoS One. 2010;5:e15647.

S42. Chen C, Li Z, Yang Y, Xiang T, Song W, Liu S. Microarray expression profiling of dysregulated long non-coding RNAs in triple-negative breast cancer. Cancer Biol Ther. 2015;16:856-65.

S43. Lehmann-Che J, Hamy AS, Porcher R, Barritault M, Bouhidel F, Habuellelah H et al. Molecular apocrine breast cancers are aggressive estrogen negative tumors overexpressing either HER2 or GCDFP15. Breast Cancer Res. 2013;15:R37.

S44. Kassambara A, Rème T, Jourdan M, Fest T, Hose D, Tarte K et al. GenomicScape : an easy-to-use web tool for gene expression data analysis. Application to investigate the molecular events in the differentiation of B cells into plasma cells. PLoS Comput Biol. 2015;11:e1004077.
