# Supplementary material for: Identification of three subtypes of triple-negative breast cancer with potential therapeutic implications
Source: Breast Cancer Res. 2019 May 17;21:65. doi: 10.1186/s13058-019-1148-6 (PMC6525459; doi:10.1186/s13058-019-1148-6)
Supplement: Supplementary file 9 — Categorical GES analyses result in function of internal TNBC clusters (C1, C2, and C3). (PDF 107 kb) [file 13058_2019_1148_MOESM9_ESM.pdf]

Additional file 9: Categorical GES analyses results in function of internal TNBC clusters (C1, C2 and C3).

GES name	subtype	All (*n* = 238)	C1 (*n* = 55)	C2 (*n* = 98)	C3 (*n* = 85)	*P*
4-TNBC	BLIA	140	12	46	82	< 0.0001
	BLIS	51	0	49	2	
	LAR	33	33	0	0	
	MES	14	10	3	1	
	Unclassified	0	0	0	0	
CIT	Basal-like	181	2	97	82	< 0.0001
	Luminal A	0	0	0	0	
	Luminal B	0	0	0	0	
	Luminal C	6	6	0	0	
	Molecular-apocrine	27	26	0	1	
	Normal	11	11	0	0	
	Unclassified	13	10	1	2	
Claudin-low	Claudin-low	19	2	7	10	0.204
	Other	219	53	91	75	
ER-negative	CC+	22	0	22	0	< 0.0001
	CC+/IR+	160	4	72	84	
	ECM+	2	1	1	0	
	IR+	4	3	0	1	
	SR+	29	29	0	0	
	Unclassified	21	18	3	0	
PAM50	Basal-like	178	4	94	80	< 0.0001
	HER2-E	0	0	0	0	
	Luminal A	10	10	0	0	
	Luminal B	35	30	1	4	
	NBL	11	7	3	1	
	Unclassified	4	4	0	0	
TNBCtype	BL1	46	0	30	16	< 0.0001
	BL2	18	7	5	6	
	IM	50	2	1	47	
	LAR	20	20	0	0	
	M	43	0	43	0	
	MSL	16	12	1	3	
	Unclassified	45	14	18	13	

GES: gene-expression signature; BLIA: basal-like immune activated; BLIS: basal-like immune suppressed ; LAR: luminal androgen receptor; MES: mesenchymal; CIT: tumor identity card; CC+: cell cycle; CC+/IR+: cell cycle and immune response; ECM+: extracellular matrix; IR+: immune response; SR+: steroid hormone response; NBL: normal breast-like; BL1: basal-like 1; BL2: basal-like 2; IM: immunomodulatory; M: mesenchymal-like; MSL: mesenchymal stem-like.
