# Supplementary material for: Identification of three subtypes of triple-negative breast cancer with potential therapeutic implications
Source: Breast Cancer Res. 2019 May 17;21:65. doi: 10.1186/s13058-019-1148-6 (PMC6525459; doi:10.1186/s13058-019-1148-6)
Supplement: Supplementary file 10 — Continuous score GES analyses result in function of internal TNBC clusters (C1, C2, and C3). (PDF 176 kb) [file 13058_2019_1148_MOESM10_ESM.pdf]

**Additional file 10: Continuous score GES analyses results in function of internal TNBC clusters (C1, C2 and C3).**

| GES name | *P* | *P* | | | Results |
|---|---|---|---|---|---|
| | | C1 vs C2 | C1 vs C3 | C2 vs C3 | |
| **Molecular subtyping** | | | | | |
| AR | < 0.0001 | < 0.0001 | < 0.0001 | 0.1155 | C1 > C2 ≈ C3 |
| Basal-like | < 0.0001 | < 0.0001 | < 0.0001 | < 0.0001 | C1 < C3 < C2 |
| Claudin-CD24 | 0.4216 | | | | NS |
| ER | < 0.0001 | < 0.0001 | < 0.0001 | 0.4577 | C1 > C2 ≈ C3 |
| ERBB2 | 0.0010 | 0.0168 | 0.0007 | 0.4582 | C1 > C2 ≈ C3 |
| Molecular-apocrine | < 0.0001 | < 0.0001 | < 0.0001 | 0.5118 | C1 > C2 ≈ C3 |
| **Immune response** | | | | | |
| B-cell | < 0.0001 | 0.0061 | < 0.0001 | < 0.0001 | C2 < C1 < C3 |
| CYT | < 0.0001 | 0.0010 | < 0.0001 | < 0.0001 | C2 < C1 < C3 |
| IL-8 | 0.5269 | | | | NS |
| M2/M1 | < 0.0001 | 0.7797 | < 0.0001 | < 0.0001 | C1 ≈ C2 > C3 |
| M2/M1 (Becker) | < 0.0001 | 0.5333 | < 0.0001 | < 0.0001 | C1 ≈ C2 > C3 |
| MHC-1 | < 0.0001 | 0.9562 | < 0.0001 | < 0.0001 | C1 ≈ C2 < C3 |
| MHC-2 | < 0.0001 | < 0.0001 | 0.0001 | < 0.0001 | C2 < C1 < C3 |
| STAT1 | < 0.0001 | 0.9341 | < 0.0001 | < 0.0001 | C3 > C1 ≈ C2 |
| T-cell | < 0.0001 | < 0.0001 | < 0.0001 | < 0.0001 | C2 < C1 < C3 |
| Type I IFN | < 0.0001 | 0.9658 | < 0.0001 | < 0.0001 | C1 ≈ C2 < C3 |
| **Microenvironment cells** | | | | | |
| Epithelial cells | 0.0731 | | | | NS |
| Fibroblasts | 0.0008 | 0.9814 | 0.0061 | 0.0022 | C1 ≈ C2 > C3 |
| Neurons | 0.0208 | 0.9967 | 0.0664 | 0.0323 | C2 > C3, C1 ≈ C2 and C1 ≈ C3 |
| **Metabolism** | | | | | |
| Adipocytes | < 0.0001 | < 0.0001 | < 0.0001 | 0.5065 | C1 > C2 ≈ C3 |
| Glycolysis | < 0.0001 | < 0.0001 | 0.0039 | 0.1663 | C1 < C2 ≈ C3 |
| IRGS | < 0.0001 | < 0.0001 | < 0.0001 | 0.0022 | C1 < C3 < C2 |
| **Critical biological pathways in cancer** | | | | | |
| AKT | < 0.0001 | 0.0003 | < 0.0001 | 0.6547 | C1 < C2 ≈ C3 |
| β-Catenin | 0.6039 | | | | NS |
| CIN | < 0.0001 | < 0.0001 | < 0.0001 | 0.2853 | C1 < C2 ≈ C3 |
| E2F3 | < 0.0001 | < 0.0001 | 0.1687 | 0.0006 | C1 ≈ C3 < C2 |
| EGFR | < 0.0001 | < 0.0001 | < 0.0001 | 0.2540 | C1 < C2 ≈ C3 |
| HOXA | < 0.0001 | < 0.0001 | < 0.0001 | 0.6825 | C1 > C2 ≈ C3 |
| MITO/OXPHOS | 0.8867 | | | | NS |
| MYC | < 0.0001 | < 0.0001 | < 0.0001 | 0.1406 | C1 < C2 ≈ C3 |
| p53 | < 0.0001 | < 0.0001 | < 0.0001 | 0.2218 | C1 > C2 ≈ C3 |
| PIK3CA | < 0.0001 | < 0.0001 | < 0.0001 | < 0.0001 | C1 > C3 > C2 |
| PNI | < 0.0001 | < 0.0001 | 0.0013 | 0.0004 | C1 < C3 < C2 |
| PRL | < 0.0001 | < 0.0001 | < 0.0001 | 0.6417 | C1 > C2 ≈ C3 |
| Proliferation | < 0.0001 | < 0.0001 | < 0.0001 | 0.6327 | C1 < C2 ≈ C3 |
| PTEN loss | < 0.0001 | < 0.0001 | < 0.0001 | 0.9444 | C1 < C2 ≈ C3 |
| RAS | 0.1017 | | | | NS |
| Reactive stroma | < 0.0001 | < 0.0001 | < 0.0001 | 0.9429 | C1 > C2 ≈ C3 |
| SRC | 0.2019 | | | | NS |
| Stroma-CD10 | 0.0001 | 0.6269 | 0.0004 | 0.0017 | C1 ≈ C2 > C3 |
| TGFβ | 0.0113 | 0.0300 | 0.9409 | 0.0332 | C1 ≈ C3 < C2 |
| VEGF | < 0.0001 | < 0.0001 | 0.6654 | < 0.0001 | C1 ≈ C3 < C2 |
| Wound-response | < 0.0001 | < 0.0001 | < 0.0001 | 0.1877 | C1 < C2 ≈ C3 |
| YAP1-WWTR1 | 0.0069 | 0.0258 | 0.9782 | 0.0183 | C1 ≈ C3 < C2 |
| **Prognosis** | | | | | |
| 38-GES | < 0.0001 | < 0.0001 | < 0.0001 | < 0.0001 | C1 < C3 < C2 |
| 70-GES | < 0.0001 | < 0.0001 | < 0.0001 | 0.0122 | C1 < C3 < C2 |
| GGI | < 0.0001 | < 0.0001 | < 0.0001 | 0.9126 | C1 < C2 ≈ C3 |

GES: gene-expression signature; NS: not significant ($P > 0.05$).
