# Supplementary material for: Identification of three subtypes of triple-negative breast cancer with potential therapeutic implications
Source: Breast Cancer Res. 2019 May 17;21:65. doi: 10.1186/s13058-019-1148-6 (PMC6525459; doi:10.1186/s13058-019-1148-6)

Additional file 11: Significant EFS analyses based on GES scores. (A) Internal and **(B)** external C2 and C3 TNBC patients.

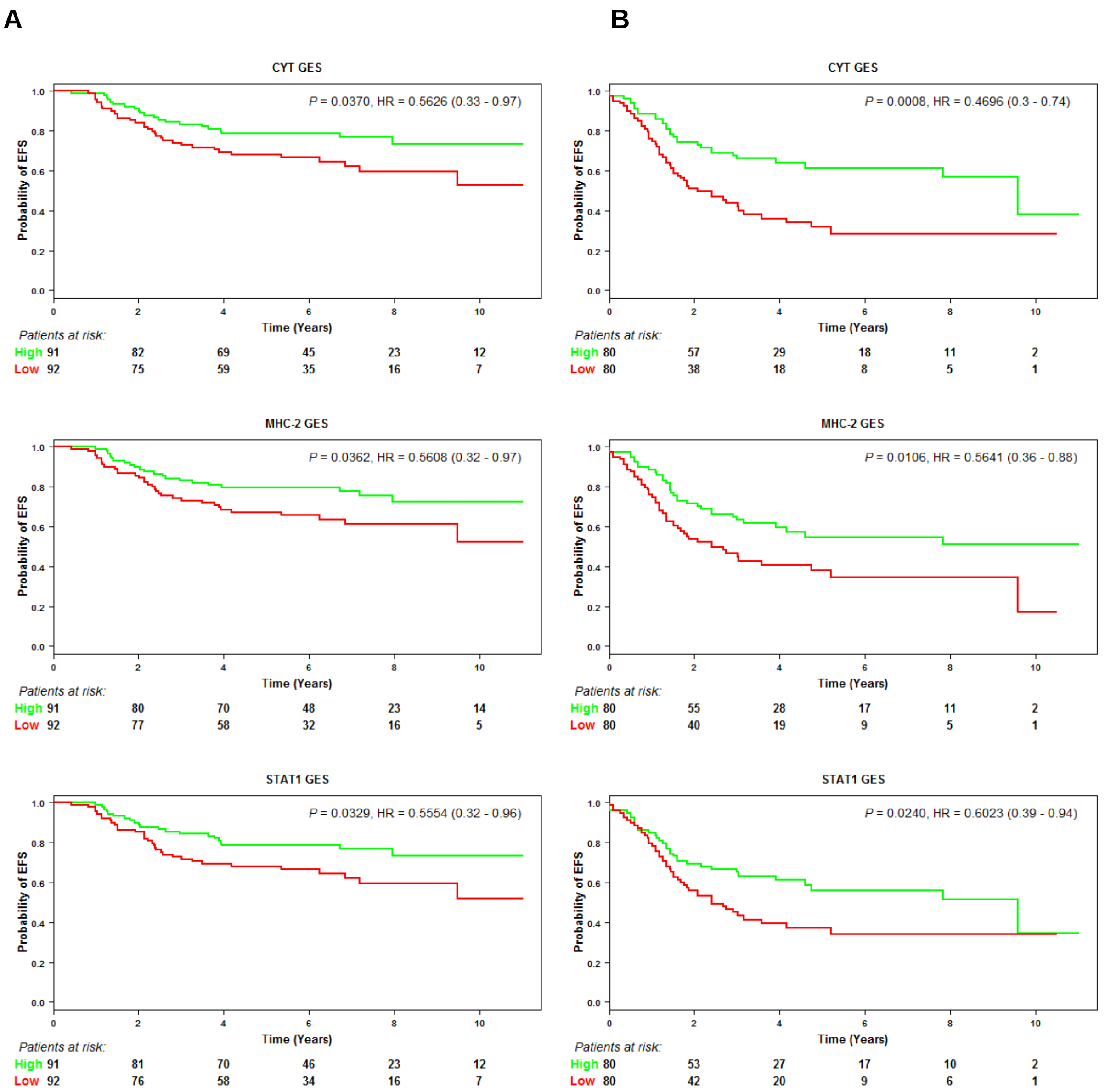

Supplement: Supplementary file 11 — Significant EFS analyses based on GES scores. (A) Internal and (B) external C2 and C3 TNBC patients. (PDF 103 kb) [file 13058_2019_1148_MOESM11_ESM.pdf]
