# Supplementary material for: Identification of three subtypes of triple-negative breast cancer with potential therapeutic implications
Source: Breast Cancer Res. 2019 May 17;21:65. doi: 10.1186/s13058-019-1148-6 (PMC6525459; doi:10.1186/s13058-019-1148-6)
Supplement: Supplementary file 12 — ToppGene GOEA based on heatmap gene clusters, internal cohort (1843 probes) and external cohorts (454 probes). Identical GO terms per cluster of highly expressed genes are indicated in bold characters. (PDF 143 kb) [file 13058_2019_1148_MOESM12_ESM.pdf]

Additional file 12: ToppGene GOEA based on heatmap gene clusters, internal cohort (1843 probes) and external cohort (454 probes). Identical GO terms per cluster of highly expressed genes are indicated in bold characters.

Cluster	Internal cohort	Cluster	External cohort
H1	Chemical homeostasis	H'1	**Response to lipid**
	**Regulation of hormone levels**		Growth
	**Hormone metabolic process**		**Hormone metabolic process**
	**Response to lipid**		Leukocyte migration
	Oxidation-reduction process		Regulation of response to wounding
	Lipid metabolic process		Regulation of response to external stimulus
	Response to steroid hormone		Chemokine production
	Digestion		Regulation of inflammatory response
	Homeostatic process		Negative regulation of cell proliferation
	**Response to hormone**		**Regulation of hormone levels**
H2a	Central nervous system development	H'2b	Embryonic eye morphogenesis
	Animal organ morphogenesis		**Sensory organ morphogenesis**
	Neurogenesis		Eye morphogenesis
	**Sensory organ development**		Forebrain neuron differentiation
	Ear development		**Sensory organ development**
	Inner ear development		Forebrain generation of neurons
	Generation of neurons		Odontogenesis of dentin-containing tooth
	**Sensory organ morphogenesis**		
	Neuron differentiation		
	Neuron development		
H2b	Antibacterial peptide production	H'2a	**Collagen catabolic process**
	Antimicrobial peptide production		Stem cell differentiation
	Neural crest cell migration		**Multicellular organismal catabolic process**
	Extracellular matrix organization		**Epithelium development**
	**Epithelium development**		Intermediate filament cytoskeleton organization
	Extracellular structure organization		Mesenchyme development
	**Collagen catabolic process**		Intermediate filament-based process
	Epidermis development		Cell motility
	**Multicellular organism catabolic process**		Localization of cell
	Ameboidal-type cell migration		Central nervous system development
H3	**Regulation of immune system process**	H'3	**Regulation of immune system process**
	**Immune response**		**Immune response**
	**Positive regulation of immune system process**		**Positive regulation of immune system process**
	**B cell receptor signaling pathway**		Antigen receptor-mediated signaling pathway
	Defense response		**B cell receptor signaling pathway**
	Immune effector process		Immune response-activating cell surface receptor signaling pathway
	**Adaptive immune response**		Immune response-regulating cell surface receptor signaling pathway
	Cell activation		Regulation of immune response
	Regulation of leucocyte activation		**Adaptive immune response**
	B cell activation		Regulation of B cell activation
