# Supplementary material for: Identification of three subtypes of triple-negative breast cancer with potential therapeutic implications
Source: Breast Cancer Res. 2019 May 17;21:65. doi: 10.1186/s13058-019-1148-6 (PMC6525459; doi:10.1186/s13058-019-1148-6)
Supplement: Supplementary file 13 — ToppGene GOEA based on SAM gene lists for the three clusters, internal and external cohorts. Identical GO terms per SAM lists are indicated in bold characters. (PDF 94 kb) [file 13058_2019_1148_MOESM13_ESM.pdf]

Additional file 13: ToppGene GOEA based on SAM gene lists for the three clusters, internal and external cohorts. Identical GO terms per SAM lists are indicated in bold characters.

	Internal cohort	External cohort
C1 high vs C2 low	**Lipid metabolic process**	**Lipid metabolic process**
	**Single-organism catabolic process**	Cell migration
	Oxidation-reduction process	**Circulatory system development**
	**Vasculature development**	**Cardiovascular system development**
	**Blood vessel development**	**Cellular lipid metabolic process**
	**Cardiovascular system development**	**Single-organism catabolic process**
	**Circulatory system development**	**Vasculature development**
	**Cellular lipid metabolic process**	Regulation of cell migration
	Secretion	**Blood vessel development**
	Organonitrogen compound catabolic process	Localization of cell
C1 low vs C2 high	**Cell cycle**	**Mitotic cell cycle**
	**Cell cycle process**	**Mitotic cell cycle process**
	**Mitotic cell cycle**	**Cell cycle**
	**Mitotic cell cycle process**	**Cell cycle process**
	**Chromosome organization**	**Mitotic nuclear division**
	**Mitotic nuclear division**	**Nuclear division**
	**Nuclear division**	**Organelle fission**
	**Organelle fission**	**Sister chromatid segregation**
	Chromosome segregation	Cell division
	**Sister chromatid segregation**	**Chromosome organization**
C1 high vs C3 low	Cardiovascular system development	Lipid metabolic process
	Circulatory system development	Cellular lipid metabolic process
	**Cell projection organization**	Lipid modification
	Movement of cell or subcellular component	**Cell projection organization**
	Anatomical structure formation involved in morphogenesis	Neuron projection development
	Cellular component morphogenesis	Fatty acid metabolic process
	Skeletal system development	Establishment of vesicle localization
	Cell morphogenesis	Lipid biosynthetic process
	Urogenital system development	Vesicle localization
	Regulation of anatomical structure morphogenesis	Neuron development
C1 low vs C3 high	**Immune response**	**Immune response**
	Mitotic cell cycle	**Lymphocyte activation**
	Mitotic cell cycle process	**Regulation of immune system process**
	Cell cycle process	**Leukocyte activation**
	Cell cycle	Positive regulation of immune response
	**Regulation of immune system process**	**Regulation of immune response**
	Positive regulation of immune system process	Leukocyte cell-cell adhesion
	**Lymphocyte activation**	Cell activation
	**Leukocyte activation**	Leukocyte aggregation
	**Regulation of immune response**	Positive regulation of immune response
C2 high vs C3 low	Bone development	No results displayed
	Dorsal/ventral pattern formation	
	Neural tube development	
	Smoothened signaling pathway	
	Cell projection organization	
	Cellular component morphogenesis	
	Cell morphogenesis	
	Neural tube closure	
	Digestive system process	
	Tube closure	
C2 low vs C3 high	**Immune response**	**Immune response**
	**Regulation of immune system process**	**Regulation of immune system process**
	**Regulation of immune response**	**Regulation of immune response**
	**Defense response**	**Positive regulation of immune system process**
	**Positive regulation of immune system process**	**Defense response**
	**Leucocyte activation**	**Leukocyte activation**
	**Cell activation**	**Cell activation**
	**Innate immune response**	**Positive regulation of immune response**
	**Lymphocyte activation**	**Innate immune response**
	**Positive regulation of immune response**	**Lymphocyte activation**
C1 high vs C2-C3 low	Cardiovascular system development	**Lipid metabolic process**
	Circulatory system development	**Cellular lipid metabolic process**
	Oxidation-reduction process	Single-organism catabolic process
	**Lipid metabolic process**	Cell migration
	Vasculature development	Neuron development
	Blood vessel development	Regulation of anatomical structure morphogenesis
	Response to endogenous stimulus	Neuron projection development
	Enzyme linked receptor protein signaling pathway	Localization of cell
	**Cellular lipid metabolic process**	Cell motility
	Organic acid catabolic process	Nucleoside diphosphate catabolic process
C1 low vs C2-C3 high	**Mitotic cell cycle**	**Mitotic cell cycle**
	**Cell cycle**	**Mitotic cell cycle process**
	**Mitotic cell cycle process**	**Cell cycle**
	**Cell cycle process**	**Cell cycle process**
	Chromosome organization	**Mitotic nuclear division**
	**Mitotic nuclear division**	**Nuclear division**
	**Nuclear division**	**Organelle fission**
	**Organelle fission**	Cell cycle phase transition
	**Sister chromatid segregation**	Mitotic cell cycle phase transition
	Chromosome segregation	**Sister chromatid segregation**
