# Supplementary material for: Identification of three subtypes of triple-negative breast cancer with potential therapeutic implications
Source: Breast Cancer Res. 2019 May 17;21:65. doi: 10.1186/s13058-019-1148-6 (PMC6525459; doi:10.1186/s13058-019-1148-6)
Supplement: Supplementary file 14 — B lymphocyte lineage GenomicScape analysis in function of the three H3 subclusters (H3a, H3b, H3c). Genes upregulated in the given group compared to the overall mean expression are highlighted in red. Genes downregulated in the given group compared to the overall mean expression are highlighted in blue. (PDF 149 kb) [file 13058_2019_1148_MOESM14_ESM.pdf]

Additional file 14: B lymphocyte lineage GenomicScape analysis in function of the three H3 subclusters (H3a, H3b, H3c). Genes upregulated in the given group compared to the overall mean expression are highlighted in red. Genes downregulated in the given group compared to the overall mean expression are highlighted in blue.

Cluster	Affymetrix probe set ID	Gene Symbol na35	Score	NBCs	CBs	CCs	MBCs	prePBs	PBs	EPCs	BMPCs
H3a	227915_at	*ASB2*	0.13	-1.80	3.27	4.85	-1.97	2.35	-1.37	-2.86	-0.85
	220423_at	*PLA2G2D*	0.20	-3.41	-3.41	-3.41	-3.41	-3.41	-3.31	-3.41	22.40
	210321_at	*GZMH*	0.14	-1.43	-1.43	-1.43	-1.22	-1.43	7.90	-1.37	-0.165
	1555120_at	*CD96*	0.11	-1.05	-1.05	-1.05	-1.05	6.95	-1.05	-1.05	-1.05
	210164_at	*GZMB*	0.13	-1.68	-1.74	-1.70	-0.85	0.12	5.16	1.63	-1.63
	238581_at	*GBP5*	0.15	2.08	-4.33	-3.46	0.91	0.52	2.53	3.13	-2.925
	219836_at	*ZBED2*	0.19	-2.56	-0.53	6.52	-2.56	8.035	-2.56	-2.56	-2.56
H3b	210313_at	*LILRA4*	0.06	-0.05	-0.91	-0.34	1.60	-1.44	-0.49	-0.23	1.61
	1558186_s_at	---	0.16	-2.79	5.70	5.395	-2.81	-2.78	-0.34	2.61	-2.76
	235885_at	*P2RY12*	0.21	-2.04	6.80	8.74	-2.21	-2.295	-2.29	-2.28	-1.31
	1553380_at	*PARP15*	0.22	7.42	-4.64	-4.35	6.915	4.10	-2.37	-4.37	-4.495
	235982_at	*FCRL1*	0.225	6.79	3.35	3.79	4.02	-2.95	-4.88	-4.88	-3.825
	228592_at	*MS4A1*	0.26	4.49	4.39	5.00	4.37	-0.56	-7.54	-7.43	-0.83
	206255_at	*BLK*	0.15	3.21	2.47	1.72	3.17	-1.21	-0.64	-3.08	-4.815
	235401_s_at	*FCRLA*	0.09	-0.11	2.59	3.02	-0.45	0.09	-1.26	-1.90	-0.85
	206398_s_at	*CD19*	0.11	1.45	2.99	3.58	0.69	-0.31	-1.55	-2.98	-2.56
	205544_s_at	*CR2*	0.16	1.13	4.95	5.85	-0.49	-3.24	-2.72	-0.30	-3.015
	224499_s_at	*AICDA*	0.34	-5.79	15.62	10.62	-5.79	7.70	-5.79	-5.79	-5.55
	230896_at	*BEND4*	0.145	2.19	2.25	2.08	2.30	1.89	-1.33	-4.52	-3.98
H3c	217260_x_at	*LOC102723479*	0.05	-0.78	-0.79	-0.65	-0.80	0.22	0.06	1.32	1.13
	230673_at	*PKHD1L1*	0.06	-1.78	1.30	1.23	-1.42	-0.62	1.01	0.77	0.02
	242020_s_at	*ZBP1*	0.14	0.83	-3.83	-3.71	1.47	-3.07	1.32	2.78	2.71
	216829_at	---	0.17	-2.82	-2.85	0.39	-3.985	-2.37	2.19	4.34	4.60
	217235_x_at	*IGLL5*	0.12	-3.16	-1.37	0.32	-2.74	-0.37	2.09	2.46	2.54
	211637_x_at	---	0.14	-1.14	-2.29	0.69	-4.41	-1.23	2.28	2.65	3.13
	211639_x_at	---	0.135	-0.67	-3.16	0.65	-4.30	-1.40	2.71	3.17	2.50
	219159_s_at	*SLAMF7*	0.23	-5.90	-4.29	0.27	-5.13	0.09	3.59	4.72	5.85
	211650_x_at	---	0.13	-1.94	-2.72	0.42	-3.87	-0.39	2.27	2.98	2.78
	211908_x_at	*IGK*	0.145	-2.28	-3.28	0.455	-3.93	-0.46	2.51	3.12	3.30
	217281_x_at	---	0.12	-1.82	-2.15	0.79	-3.73	-0.46	2.20	2.02	2.88
	217022_s_at	---	0.12	-5.16	-2.68	-0.39	0.08	1.495	2.10	2.15	1.795
	212592_at	*IGJ*	0.08	-2.90	-0.81	0.54	-2.30	0.62	2.13	2.29	0.36
	224405_at	*FCRL5*	0.12	-1.06	-1.92	0.66	-2.66	-2.31	2.29	2.045	2.72
	207237_at	*KCNA3*	0.10	1.86	-2.99	-3.37	-0.13	-0.22	1.27	1.695	0.61
	206641_at	*TNFRSF17*	0.22	-7.31	0.635	1.46	-5.69	1.73	3.27	3.23	3.09
	223565_at	*MZB1*	0.14	-2.59	-1.17	0.89	-4.52	-0.56	2.18	2.22	3.50

NBCs: naive B cells; CBs: centroblasts; CCs: centrocytes; MBCs: memory B cells; prePBS: preplasmablasts; PBs: plasmablasts; EPCs: early plasma cells; BMPCs: bone marrow plasma cells.
