# Supplementary material for: Identification of three subtypes of triple-negative breast cancer with potential therapeutic implications
Source: Breast Cancer Res. 2019 May 17;21:65. doi: 10.1186/s13058-019-1148-6 (PMC6525459; doi:10.1186/s13058-019-1148-6)

Additional file 15: Biological gradients between C2 and C3 in our internal cohort. Correlation coefficient calculation between GES score for each internal cohort tumor (x number), and C2 and C3 cluster probability orthogonal projection on C2-C3 axis (y number) (C2 tumor, red; C3 tumor, green). Only correlation coefficient absolute values superior to 0.5 ($P < 0.0001$) are displayed.

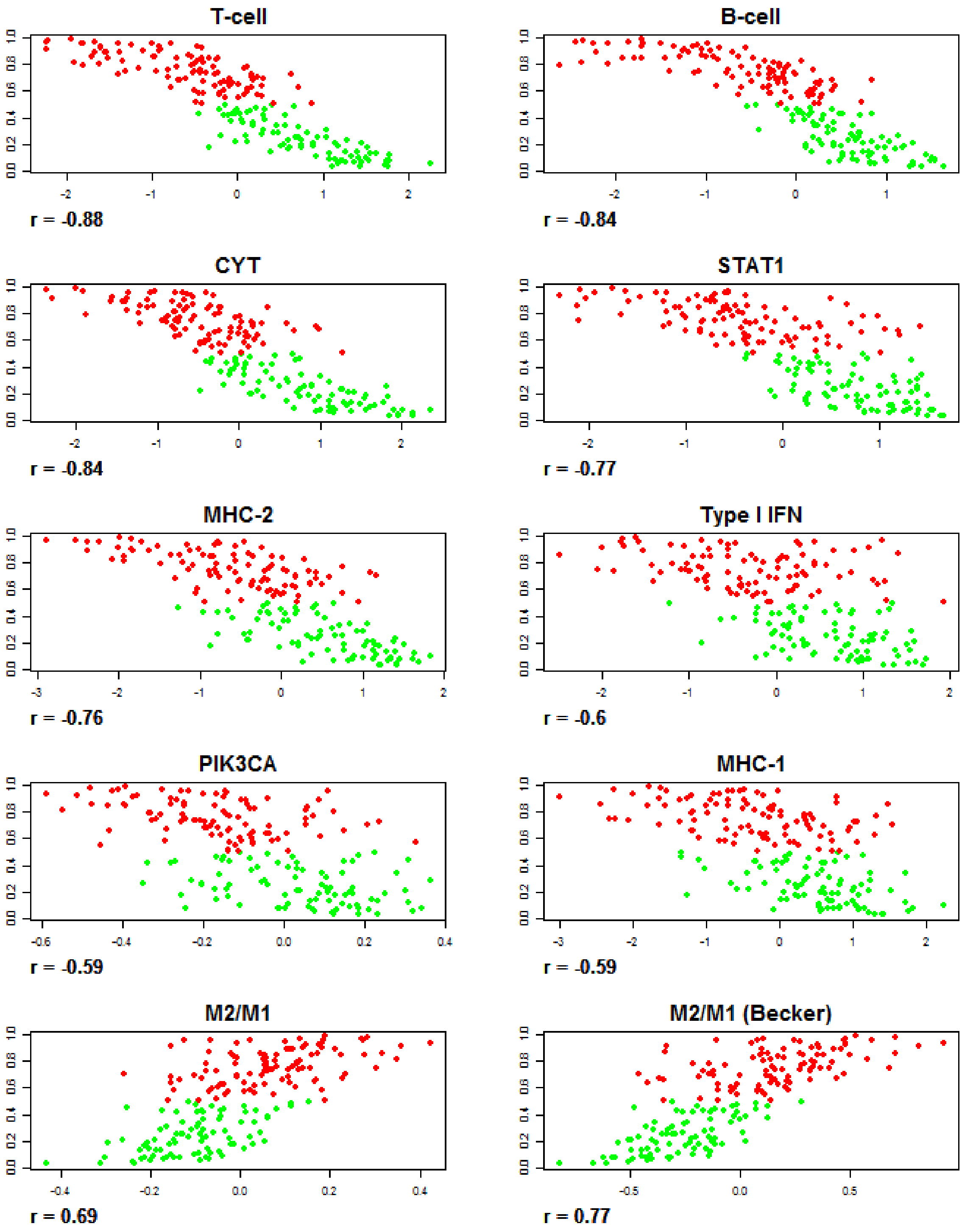

Supplement: Supplementary file 15 — Biological gradients between C2 and C3 in our internal cohort. Correlation coefficient calculation between GES score for each internal cohort tumor (x number), and C2 and C3 cluster probability orthogonal projection on C2-C3 axis (y number) (C2 tumor, red; C3 tumor, green). Only correlation coefficient absolute values superior to 0.5 (P < 0.0001) are displayed. (PDF 101 kb) [file 13058_2019_1148_MOESM15_ESM.pdf]
