# Supplementary material for: Identification of three subtypes of triple-negative breast cancer with potential therapeutic implications
Source: Breast Cancer Res. 2019 May 17;21:65. doi: 10.1186/s13058-019-1148-6 (PMC6525459; doi:10.1186/s13058-019-1148-6)
Supplement: Supplementary file 16 — Expression of immune checkpoints between basal-like clusters. Internal (C2 vs C3) and external (C’2 vs C’3) cohorts. (PDF 158 kb) [file 13058_2019_1148_MOESM16_ESM.pdf]

Additional file 16: Expression of immune checkpoints between basal-like clusters. Internal (C2 vs C3) and external (C'2 vs C'3) cohorts.

Gene symbol	*P* C2 vs C3	C2 vs C3	*P* C'2 vs C'3	C'2 vs C'3
ADORA2A	< 0.0001	2 < 3	< 0.0001	2 < 3
BTLA	< 0.0001	2 < 3	< 0.0001	2 < 3
BTNL2	0.4081	2 = 3	0.9107	2 = 3
C10orf54	< 0.0001	2 < 3	< 0.0001	2 < 3
CD27	< 0.0001	2 < 3	< 0.0001	2 < 3
CD28	< 0.0001	2 < 3	< 0.0001	2 < 3
CD40	< 0.0001	2 < 3	< 0.0001	2 < 3
CD40LG	< 0.0001	2 < 3	< 0.0001	2 < 3
CD44	0.0356	2 < 3	0.913	2 < 3
CD48	< 0.0001	2 < 3	< 0.0001	2 < 3
CD70	0.0082	2 < 3	0.0004	2 < 3
CD80	< 0.0001	2 < 3	< 0.0001	2 < 3
CD86	< 0.0001	2 < 3	< 0.0001	2 < 3
CD160	< 0.0001	2 < 3	0.0081	2 < 3
CD200	0.0005	2 < 3	0.0026	2 < 3
CD200R1	< 0.0001	2 < 3	< 0.0001	2 < 3
CD244	< 0.0001	2 < 3	0.0946	2 = 3
CD274 (PD-L1)	< 0.0001	2 < 3	< 0.0001	2 < 3
CD276	0.2043	2 = 3	0.7991	2 = 3
CTLA4	< 0.0001	2 < 3	< 0.0001	2 < 3
HAVCR2	< 0.0001	2 < 3	< 0.0001	2 < 3
HHLA2	0.4083	2 = 3	0.6122	2 = 3
ICOS	< 0.0001	2 < 3	< 0.0001	2 < 3
ICOSLG	0.5792	2 = 3	0.2997	2 = 3
IDO1	< 0.0001	2 < 3	< 0.0001	2 < 3
IDO2	< 0.0001	2 < 3	0.0001	2 < 3
KIR3DL1	0.0067	2 < 3	0.1582	2 = 3
LAG3	< 0.0001	2 < 3	< 0.0001	2 < 3
LAIR1	< 0.0001	2 < 3	< 0.0001	2 < 3
LGALS9	< 0.0001	2 < 3	< 0.0001	2 <3
NRP1	0.785	2 = 3	0.0076	2 < 3
PDCD1 (PD1)	0.0002	2 < 3	0.0002	2 < 3
PDCD1LG2	< 0.0001	2 < 3	< 0.0001	2 < 3
TIGIT	< 0.0001	2 < 3	< 0.0001	2 < 3
TMIGD2	0.1157	2 = 3	0.0063	2 < 3
TNFRSF4	< 0.0001	2 < 3	0.0001	2 < 3
TNFRSF8	0.0109	2 < 3	< 0.0001	2 < 3
TNFRSF9	< 0.0001	2 < 3	< 0.0001	2 < 3
TNFRSF14	< 0.0001	2 < 3	< 0.0001	2 < 3
TNFRSF18	< 0.0001	2 < 3	< 0.0001	2 < 3
TNFRSF25	< 0.0001	2 < 3	0.0056	2 < 3
TNFSF4	0.0001	2 < 3	< 0.0001	2 < 3
TNFSF9	0.0003	2 < 3	0.9956	2 = 3
TNFSF14	< 0.0001	2 < 3	< 0.0001	2 < 3
TNFSF15	0.7979	2 = 3	0.0005	2 < 3
TNFSF18	0.1632	2 = 3	0.8899	2 = 3
VTCN1 (B7-H4)	0.0092	2 > 3	< 0.0001	2 > 3
