# Supplementary material for: Identification of three subtypes of triple-negative breast cancer with potential therapeutic implications
Source: Breast Cancer Res. 2019 May 17;21:65. doi: 10.1186/s13058-019-1148-6 (PMC6525459; doi:10.1186/s13058-019-1148-6)
Supplement: Supplementary file 17 — Comparison of internal and external TNBC cohorts’ clinicopathologic characteristics. (PDF 135 kb) [file 13058_2019_1148_MOESM17_ESM.pdf]

Additional file 17: Comparison of internal and external TNBC cohorts' clinicopathologic characteristics.

Characteristic		Internal cohort (*n* = 238)	External cohort (*n* = 257)	*P*
Clusters				
	1	55	61	0.73
	2	98	97	
	3	85	99	
Age (years; mean ± sd)				
		54.6± 11.6	53.5± 12.3	0.34
SBR grade				
	1 or 2	43	46	1.00
	3	195	205	

sd: standard deviation
