# Supplementary material for: Identification of three subtypes of triple-negative breast cancer with potential therapeutic implications
Source: Breast Cancer Res. 2019 May 17;21:65. doi: 10.1186/s13058-019-1148-6 (PMC6525459; doi:10.1186/s13058-019-1148-6)
Supplement: Supplementary file 18 — External TNBC cohort’s clinicopathologic characteristics in function of the three clusters (C’1, C’2, and C’3). (PDF 136 kb) [file 13058_2019_1148_MOESM18_ESM.pdf]

Additional file 18: External TNBC cohort's clinicopathologic characteristics in function of the three clusters (C'1, C'2 and C'3).

**Characteristic**	**All (*n* = 257)**	**C'1 (*n* = 61)**	**C'2 (*n* = 97)**	**C'3 (*n* = 99)**	***P***
Age (years; mean± sd)					
	53.5± 12.3	59.1±11.7	52.0±12.5	51.6±11.4	0.0002
SBR grade					
1 or 2	46	19	13	14	0.0134
3	205	41	81	83	

sd: standard deviation
