# Supplementary material for: Identification of three subtypes of triple-negative breast cancer with potential therapeutic implications
Source: Breast Cancer Res. 2019 May 17;21:65. doi: 10.1186/s13058-019-1148-6 (PMC6525459; doi:10.1186/s13058-019-1148-6)
Supplement: Supplementary file 19 — Comparison of clinicopathologic characteristics of TNBC cohorts in function of clusters. Internal (C1, n = 55; C2, n = 98; C3, n = 85) and external (C’1, n = 61; C’2, n = 97; C’3, n = 99). (PDF 138 kb) [file 13058_2019_1148_MOESM19_ESM.pdf]

Additional file 19: Comparison of clinicopathologic characteristics of TNBC cohorts in function of clusters. Internal (C1, *n* = 55; C2, *n* = 98; C3, *n* = 85) and external (C'1, *n* = 61; C'2, *n* = 97; C'3, *n* = 99).

Characteristic		Internal cohort	External cohort	*P*
Age (years; mean ± sd)				
	1	58.4 ± 11.4	59.1 ± 11.7	0.73
	2	54.4 ± 11.8	52.0 ± 12.5	0.17
	3	52.3 ± 11.0	51.6 ± 11.4	0.69
SBR grade				
	1: 1 or 2	18	19	1.00
	3	37	41	
	2: 1 or 2	10	13	0.51
	3	88	81	
	3: 1 or 2	15	14	0.69
	3	70	83	

sd: standard deviation
