# Supplementary material for: Identification of three subtypes of triple-negative breast cancer with potential therapeutic implications
Source: Breast Cancer Res. 2019 May 17;21:65. doi: 10.1186/s13058-019-1148-6 (PMC6525459; doi:10.1186/s13058-019-1148-6)
Supplement: Supplementary file 20 — Categorical GES analyses result in function of external TNBC clusters (C’1, C’2 and C’3). (PDF 159 kb) [file 13058_2019_1148_MOESM20_ESM.pdf]

**Additional file 20: Categorical GES analyses results in function of external TNBC clusters (C'1, C'2 and C'3).**

| GES name | subtype | All (*n* = 257) | C'1 (*n* = 61) | C'2 (*n* = 97) | C'3 (*n* = 99) | *P* |
|---|---|---|---|---|---|---|
| 4-TNBC | BLIA | 109 | 15 | 19 | 75 | < 0.0001 |
| | BLIS | 101 | 4 | 76 | 21 | |
| | LAR | 18 | 18 | 0 | 0 | |
| | MES | 18 | 13 | 2 | 3 | |
| | Unclassified | 11 | 11 | 0 | 0 | |
| CIT | Basal-like | 187 | 5 | 89 | 93 | < 0.0001 |
| | Luminal A | 0 | 0 | 0 | 0 | |
| | Luminal B | 1 | 1 | 0 | 0 | |
| | Luminal C | 11 | 11 | 0 | 0 | |
| | Molecular-apocrine | 31 | 31 | 0 | 0 | |
| | Normal | 7 | 4 | 3 | 0 | |
| | Unclassified | 20 | 9 | 5 | 6 | |
| Claudin-low | Claudin-low | 12 | 3 | 2 | 7 | 0.2259 |
| | Other | 245 | 58 | 95 | 92 | |
| ER-negative | CC+ | 67 | 7 | 47 | 13 | < 0.0001 |
| | CC+/IR+ | 125 | 7 | 42 | 76 | |
| | ECM+ | 25 | 14 | 8 | 3 | |
| | IR+ | 16 | 9 | 0 | 7 | |
| | SR+ | 22 | 22 | 0 | 0 | |
| | Unclassified | 2 | 2 | 0 | 0 | |
| PAM50 | Basal-like | 198 | 14 | 89 | 95 | < 0.0001 |
| | HER2-E | 30 | 29 | 0 | 1 | |
| | Luminal A | 8 | 8 | 0 | 0 | |
| | Luminal B | 4 | 4 | 0 | 0 | |
| | NBL | 17 | 6 | 8 | 3 | |
| | Unclassified | 0 | 0 | 0 | 0 | |
| TNBCtype | BL1 | 43 | 0 | 26 | 17 | < 0.0001 |
| | BL2 | 26 | 8 | 5 | 13 | |
| | IM | 49 | 2 | 2 | 45 | |
| | LAR | 36 | 35 | 0 | 1 | |
| | M | 39 | 1 | 37 | 1 | |
| | MSL | 19 | 8 | 8 | 3 | |
| | Unclassified | 45 | 7 | 19 | 19 | |

GES: gene-expression signature; BLIA: basal-like immune activated; BLIS: basal-like immune suppressed ; LAR: luminal androgen receptor; MES: mesenchymal; CIT: tumor identity card; CC+: cell cycle; CC+/IR+: cell cycle and immune response; ECM+: extracellular matrix; IR+: immune response; SR+: steroid hormone response; NBL: normal breast-like; BL1: basal-like 1; BL2: basal-like 2; IM: immunomodulatory; M: mesenchymal-like; MSL: mesenchymal stem-like.
