# Supplementary material for: Identification of three subtypes of triple-negative breast cancer with potential therapeutic implications
Source: Breast Cancer Res. 2019 May 17;21:65. doi: 10.1186/s13058-019-1148-6 (PMC6525459; doi:10.1186/s13058-019-1148-6)
Supplement: Supplementary file 21 — Continuous score GES analyses results in function of external TNBC clusters (C’1, C’2, and C’3). (PDF 228 kb) [file 13058_2019_1148_MOESM21_ESM.pdf]

Additional file 21: Continuous score GES analyses results in function of external TNBC clusters (C'1, C'2 and C'3).

GES name	*P*	*P*			Results
		C'1 vs C'2	C'1 vs C'3	C'2 vs C'3	
**Molecular subtyping**					
AR	< 0.0001	< 0.0001	< 0.0001	0.2924	C'1 > C'2 ≈ C'3
Basal-like	< 0.0001	< 0.0001	< 0.0001	< 0.0001	C'1 < C'3 < C'2
Claudin-CD24	0.2730				NS
ER	< 0.0001	< 0.0001	< 0.0001	0.8448	C'1 > C'2 ≈ C'3
ERBB2	< 0.0001	< 0.0001	< 0.0001	0.8803	C'1 > C'2 ≈ C'3
Molecular-apocrine	< 0.0001	< 0.0001	< 0.0001	0.8759	C'1 > C'2 ≈ C'3
**Immune response**					
B-cell	< 0.0001	0.0280	< 0.0001	< 0.0001	C'2 < C'1 < C'3
CYT	< 0.0001	0.5195	< 0.0001	< 0.0001	C'1 ≈ C'2 < C'3
IL-8	0.0434	0.3053	0.0331	0.4638	C'1 < C'3, C'1 ≈ C'2 and C'2 ≈ C'3
M2/M1	< 0.0001	0.1559	< 0.0001	< 0.0001	C'1 ≈ C'2 > C'3
M2/M1 (Becker)	< 0.0001	0.3478	< 0.0001	< 0.0001	C'1 ≈ C'2 > C'3
MHC-1	< 0.0001	0.9380	< 0.0001	< 0.0001	C'1 ≈ C'2 < C'3
MHC-2	< 0.0001	0.0026	< 0.0001	< 0.0001	C'2 < C'1 < C'3
STAT1	< 0.0001	0.9361	< 0.0001	< 0.0001	C'1 ≈ C'2 < C'3
T-cell	< 0.0001	0.0217	< 0.0001	< 0.0001	C'2 < C'1 < C'3
Type I IFN	< 0.0001	0.4534	< 0.0001	< 0.0001	C'1 ≈ C'2 < C'3
**Microenvironment cells**					
Epithelial cells	0.1900				NS
Fibroblasts	0.0077	0.5336	0.0085	0.0753	C'1 > C'3, C'1 ≈ C'2 and C'2 ≈ C'3
Neurons	0.0008	0.9737	0.0121	0.0014	C'1 ≈ C'2 > C'3
**Metabolism**					
Adipocytes	0.1683				NS
Glycolysis	0.0169	0.0628	0.0153	0.8225	C'1 < C'3, C'1 ≈ C'2 and C'2 ≈ C'3
IRGS	< 0.0001	< 0.0001	< 0.0001	0.8684	C'1 < C'2 ≈ C'3
**Critical biological pathways in cancer**					
AKT	0.2110				NS
β-Catenin	0.1171				NS
CIN	< 0.0001	< 0.0001	< 0.0001	0.9767	C'1 < C'2 ≈ C'3
E2F3	0.0014	0.0082	0.9831	0.0039	C'1 ≈ C'3 < C'2
EGFR	< 0.0001	< 0.0001	< 0.0001	0.4994	C'1 < C'2 ≈ C'3
HOXA	0.0001	0.0231	< 0.0001	0.1015	C'1 > C'2 ≈ C'3
MITO/OXPHOS	0.1581				NS
MYC	< 0.0001	< 0.0001	< 0.0001	0.4907	C'1 < C'2 ≈ C'3
p53	< 0.0001	< 0.0001	< 0.0001	0.0697	C'1 > C'2 ≈ C'3
PIK3CA	< 0.0001	< 0.0001	< 0.0001	< 0.0001	C'1 < C'3 < C'2
PNI	0.0002	0.0005	0.5177	0.0052	C'1 ≈ C'3 < C'2
PRL	< 0.0001	< 0.0001	0.0027	0.2571	C'1 > C'2 ≈ C'3
Proliferation	< 0.0001	< 0.0001	< 0.0001	0.8548	C'1 < C'2 ≈ C'3
PTEN loss	< 0.0001	< 0.0001	< 0.0001	0.9473	C'1 < C'2 ≈ C'3
RAS	0.0016	0.1729	0.3544	0.001	C'2 < C'3, C'1 ≈ C'2 and C'1 ≈ C'3
Reactive stroma	< 0.0001	< 0.0001	< 0.0001	0.3142	C'1 > C'2 ≈ C'3
SRC	0.0001	0.0682	< 0.0001	0.0519	C'1 < C'3, C'1 ≈ C'2 and C'2 ≈ C'3
Stroma-CD10	< 0.0001	< 0.0001	< 0.0001	0.8734	C'1 > C'2 ≈ C'3
TGFβ	0.0507				NS
VEGF	0.0130	0.1321	0.8256	0.0119	C'2 > C'3, C'1 ≈ C'2 and C'1 ≈ C'3
Wound-response	< 0.0001	< 0.0001	< 0.0001	0.7747	C'1 < C'2 ≈ C'3
YAP1-WWTR1	0.9617				NS
**Prognosis**					
38-GES	< 0.0001	< 0.0001	< 0.0001	0.1054	C'1 < C'2 ≈ C'3
70-GES	< 0.0001	< 0.0001	< 0.0001	0.0391	C'1 < C'3 < C'2
GGI	< 0.0001	< 0.0001	< 0.0001	0.5274	C'1 < C'2 ≈ C'3

GES: gene-expression signature; NS: not significant (p > 0.05).
