# Supplementary material for: Identification of three subtypes of triple-negative breast cancer with potential therapeutic implications
Source: Breast Cancer Res. 2019 May 17;21:65. doi: 10.1186/s13058-019-1148-6 (PMC6525459; doi:10.1186/s13058-019-1148-6)
Supplement: Supplementary file 22 — C’2 vs C’3 CIBERSORT analysis results. (PDF 199 kb) [file 13058_2019_1148_MOESM22_ESM.pdf]

Additional file 22: C’2 vs C’3 CIBERSORT analysis results.

**Immune cells**	**Tumorigenic effect in breast cancer[a]**	***P***	**C’2 vs C’3 differential expression**
B cells memory	Pro-tumorigenic	0.0825	C’2 ≈ C’3
B cells naïve	No effect	0.4607	C’2 ≈ C’3
Dendritic cells activated	Anti-tumorigenic	0.4391	C’2 ≈ C’3
Dendritic cells resting	Pro-tumorigenic	0.9313	C’2 ≈ C’3
Eosinophils	Pro-tumorigenic	0.2689	C’2 ≈ C’3
Macrophages M0	Pro-tumorigenic	< 0.0001	C’2 > C’3
Macrophages M1	Pro-tumorigenic	0.0140	C’2 < C’3
Macrophages M2	Pro-tumorigenic	0.1686	C’2 ≈ C’3
Mast cells activated	Pro-tumorigenic	0.0075	C’2 > C’3
Mast cells resting	Anti-tumorigenic	0.3859	C’2 ≈ C’3
Monocytes	Pro-tumorigenic	0.4888	C’2 ≈ C’3
Neutrophils	Pro-tumorigenic	0.9263	C’2 ≈ C’3
NK cells activated	Anti-tumorigenic	0.3807	C’2 ≈ C’3
NK cells resting	Pro-tumorigenic	0.1304	C’2 ≈ C’3
Plasma cells	Anti-tumorigenic	0.0097	C’2 < C’3
T cells CD4 memory activated	Anti-tumorigenic	< 0.0001	C’2 < C’3
T cells CD4 memory resting	Pro-tumorigenic	0.0346	C’2 > C’3
T cells CD4 naive	Anti-tumorigenic	0.3192	C’2 ≈ C’3
T cells CD8	Anti-tumorigenic	< 0.0001	C’2 < C’3
T cells follicular helper	Anti-tumorigenic	0.9388	C’2 ≈ C’3
T cells gamma delta	Anti-tumorigenic	0.2758	C’2 ≈ C’3
T cells regulatory	Anti-tumorigenic	0.2276	C’2 ≈ C’3

a: based on results from Gentles et al [21] except for macrophages M1 [22]
