# Supplementary material for: Identification of three subtypes of triple-negative breast cancer with potential therapeutic implications
Source: Breast Cancer Res. 2019 May 17;21:65. doi: 10.1186/s13058-019-1148-6 (PMC6525459; doi:10.1186/s13058-019-1148-6)

Additional file 23: Biological gradients between C2 and C3 in external cohort. Correlation coefficient calculation between GES score for each external cohort tumor (x number), and C'2 and C'3 cluster probability orthogonal projection on C'2-C'3 axis (y number) (C'2 tumor, red; C'3 tumor, green). Only correlation coefficient absolute values superior to 0.5 (*P* < 0.0001) are displayed.

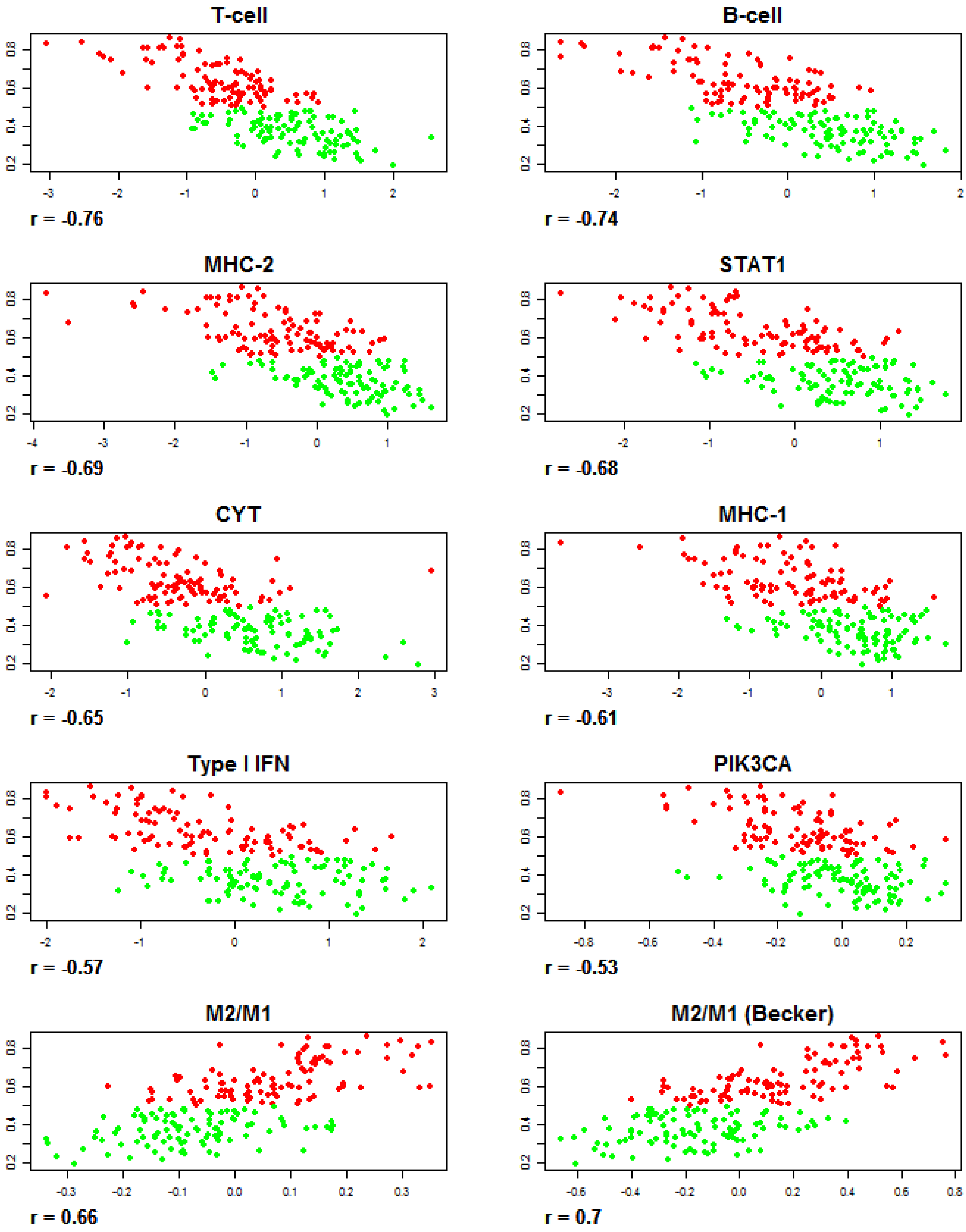

Supplement: Supplementary file 23 — Biological gradients between C2 and C3 in an external cohort. Correlation coefficient calculation between GES score for each external cohort tumor (x number), and C’2 and C’3 cluster probability orthogonal projection on C’2-C’3 axis (y number) (C’2 tumor, red; C’3 tumor, green). Only correlation coefficient absolute values superior to 0.5 (P < 0.0001) are displayed. (PDF 155 kb) [file 13058_2019_1148_MOESM23_ESM.pdf]
