# Supplementary material for: Identification of three subtypes of triple-negative breast cancer with potential therapeutic implications
Source: Breast Cancer Res. 2019 May 17;21:65. doi: 10.1186/s13058-019-1148-6 (PMC6525459; doi:10.1186/s13058-019-1148-6)

Additional file 24: PCA result representation of external TNBC and non-TNBC data. PCA is computed based on the TNBC data and the values of the two first principal components (PC1 and PC2) are then predicted for the non-TNBC data. All patients are then projected onto the plane generated by PC1 and PC2. Ellipses represent the 95% confidence of belonging to the clusters data (C'1, *n* = 61 [blue]; C'2, *n* = 97 [red] and C'3, *n* = 99 [green]). External non-TNBC data (n = 894) are figured by black points.

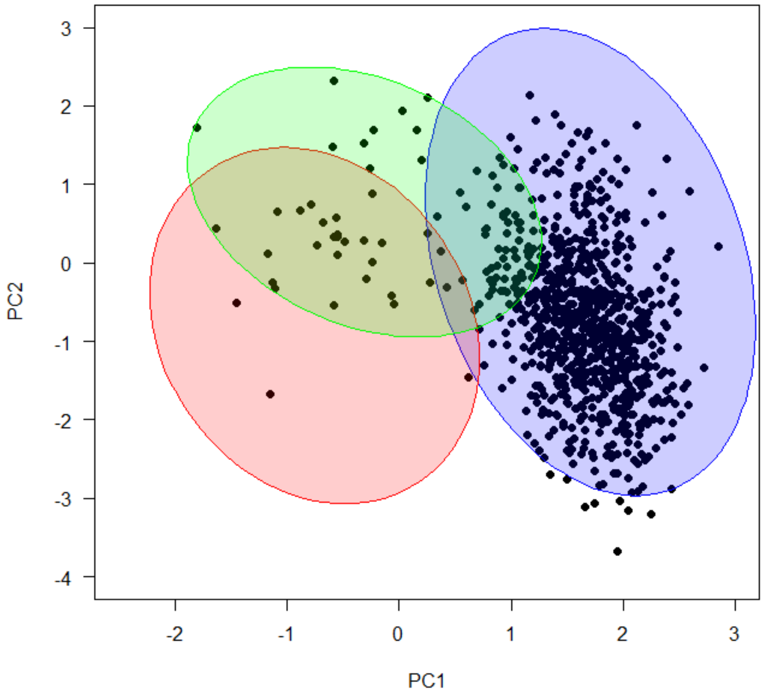

Supplement: Supplementary file 24 — PCA result representation of external TNBC and non-TNBC data. PCA is computed based on the TNBC data and the values of the two first principal components (PC1 and PC2) are then predicted for the non-TNBC data. All patients are then projected onto the plane generated by PC1 and PC2. Ellipses represent the 95% confidence of belonging to the clusters data (C’1, n = 61 [blue]; C’2, n = 97 [red] and C’3, n = 99 [green]). External non-TNBC data (n = 894) are figured by black points. (PDF 149 kb) [file 13058_2019_1148_MOESM24_ESM.pdf]
