# Supplementary material for: Identification of three subtypes of triple-negative breast cancer with potential therapeutic implications
Source: Breast Cancer Res. 2019 May 17;21:65. doi: 10.1186/s13058-019-1148-6 (PMC6525459; doi:10.1186/s13058-019-1148-6)
Supplement: Supplementary file 25 — Categorical GES analyses interpretation in function of external TNBC (C’1, C’2, C’3) and non-TNBC clusters (NTN). (PDF 238 kb) [file 13058_2019_1148_MOESM25_ESM.pdf]

Additional file 25: Categorical GES analyses interpretation in function of external TNBC (C'1, C'2, C'3) and non-TNBC clusters (NTN).

GES name	subtype	All (*n* = 1151)	NTN (*n* = 894)	C'1 (*n* = 61)	C'2 (*n* = 97)	C'3 (*n* = 99)	*P*
4-TNBC	BLIA	109		15	19	75	< 0.0001
	BLIS	101		4	76	21	
	LAR	18		18	0	0	
	MES	18		13	2	3	
	Unclassified	11		11	0	0	
CIT	Basal-like	225	38	5	89	93	< 0.0001
	Luminal A	148	148	0	0	0	
	Luminal B	165	164	1	0	0	
	Luminal C	142	131	11	0	0	
	Molecular-apocrine	96	65	31	0	0	
	Normal	192	185	4	3	0	
	Unclassified	183	163	9	5	6	
Claudin-low	Claudin-low	12		3	2	7	0.2259
	Other	245		58	95	92	
ER-negative	CC+	156	89	7	47	13	< 0.0001
	CC+/IR+	230	105	7	42	76	
	ECM+	301	276	14	8	3	
	IR+	151	135	9	0	7	
	SR+	266	245	22	0	0	
	Unclassified	47	45	2	0	0	
PAM50	Basal-like	278	80	14	89	95	< 0.0001
	HER2-E	165	135	29	0	1	
	Luminal A	394	386	8	0	0	
	Luminal B	215	211	4	0	0	
	NBL	92	75	6	8	3	
	Unclassified	7	7	0	0	0	
TNBCtype	BL1	43		0	26	17	< 0.0001
	BL2	26		8	5	13	
	IM	49		2	2	45	
	LAR	36		35	0	1	
	M	39		1	37	1	
	MSL	19		8	8	3	
	Unclassified	45		7	19	19	

GES: gene-expression signature; BLIA: basal-like immune activated; BLIS: basal-like immune suppressed ; LAR: luminal androgen receptor; MES: mesenchymal; CIT: tumor identity card; CC+: cell cycle; CC+/IR+: cell cycle and immune response; ECM+: extracellular matrix; IR+: immune response; SR+: steroid hormone response; NBL: normal breast-like; BL1: basal-like 1; BL2: basal-like 2; IM: immunomodulatory; M: mesenchymal-like; MSL: mesenchymal stem-like.
