# Supplementary material for: Identification of three subtypes of triple-negative breast cancer with potential therapeutic implications
Source: Breast Cancer Res. 2019 May 17;21:65. doi: 10.1186/s13058-019-1148-6 (PMC6525459; doi:10.1186/s13058-019-1148-6)
Supplement: Supplementary file 26 — Continuous score GES analyses results in function of external TNBC (C’1, C’2, C’3) and non-TNBC clusters (NTN). (PDF 245 kb) [file 13058_2019_1148_MOESM26_ESM.pdf]

Additional file 26: Continuous score GES analyses results in function of external TNBC (C'1, C'2, C'3) and non-TNBC clusters (NTN).

GES name	*P*	*P*						Results
		NTN vs C'1	NTN vs C'2	NTN vs C'3	C'1 vs C'2	C'1 vs C'3	C'2 vs C'3	
**Molecular subtyping**								
AR	< 0.0001	0.5261	< 0.0001	< 0.0001	< 0.0001	< 0.0001	0.4192	NTN ≈ C'1 > C'2 ≈ C'3
Basal-like	< 0.0001	0.7887	< 0.0001	< 0.0001	< 0.0001	< 0.0001	< 0.0001	NTN ≈ C'1 < C'3 < C'2
Claudin-CD24	0.0428	0.8129	0.9223	0.0460	0.6719	0.0751	0.4628	NTN > C'3, NTN ≈ C'1 ≈ C'2 and C'1 ≈ C'2 ≈ C'3
ER	< 0.0001	< 0.0001	< 0.0001	< 0.0001	< 0.0001	< 0.0001	0.9994	NTN > C'1 > C'2 ≈ C'3
ERBB2	< 0.0001	0.9015	< 0.0001	< 0.0001	< 0.0001	< 0.0001	0.9988	NTN ≈ C'1 > C'2 ≈ C'3
Molecular-apocrine	< 0.0001	0.2860	0.0117	< 0.0001	0.0031	< 0.0001	< 0.0001	C'1 > NTN > C'2 ≈ C'3
**Immune response**								
B-cell	< 0.0001	0.3858	0.1987	< 0.0001	0.0495	< 0.0001	< 0.0001	C'3 > C'1 > C'2, NTN ≈ C'2 < C'3 and NTN ≈ C'1
CYT	< 0.0001	0.7898	0.8523	< 0.0001	0.5772	< 0.0001	< 0.0001	NTN ≈ C'1 ≈ C'2 < C'3
IL-8	< 0.0001	0.0006	< 0.0001	< 0.0001	0.6170	0.1143	0.6544	NTN < C'1 ≈ C'2 ≈ C'3
M2/M1	< 0.0001	0.0146	0.9985	< 0.0001	0.0932	< 0.0001	< 0.0001	C'1 ≈ C'2 > C'3, NTN > C'1 > C'3 and NTN ≈ C'2
M2/M1 (Becker)	< 0.0001	0.4919	0.8117	< 0.0001	0.3117	< 0.0001	< 0.0001	NTN ≈ C'1 ≈ C'2 > C'3
MHC-1	< 0.0001	0.9050	0.9917	< 0.0001	0.9838	< 0.0001	< 0.0001	NTN ≈ C'1 ≈ C'2 < C'3
MHC-2	< 0.0001	0.8756	< 0.0001	< 0.0001	0.0016	0.0001	< 0.0001	C'3 > NTN ≈ C'1 > C'2
STAT1	< 0.0001	0.3643	0.6263	< 0.0001	0.9515	< 0.0001	< 0.0001	NTN ≈ C'1 ≈ C'2 < C'3
T-cell	< 0.0001	0.6182	0.0161	< 0.0001	0.0184	< 0.0001	< 0.0001	C'3 > NTN ≈ C'1 > C'2
Type I IFN	< 0.0001	1.0000	0.3096	< 0.0001	0.6751	< 0.0001	< 0.0001	NTN ≈ C'1 ≈ C'2 < C'3
**Microenvironment cells**								
Epithelial cells	0.1773							NS
Fibroblasts	< 0.0001	0.0449	< 0.0001	< 0.0001	0.5437	0.0045	0.0978	NTN > C'1 > C'3, NTN > C'2 ≈ C'3 and C'1 ≈ C'2
Neurons	0.0001	0.7117	0.3491	0.0005	0.9962	0.0034	0.0002	NTN ≈ C'1 ≈ C'2 > C'3
**Metabolism**								
Adipocytes	< 0.0001	0.8846	0.0128	0.0004	0.5005	0.1808	0.8960	NTN > C'2 ≈ C'3, NTN ≈ C'1 and C'1 ≈ C'2 ≈ C'3
Glycolysis	< 0.0001	< 0.0001	< 0.0001	< 0.0001	0.1972	0.0749	0.9614	NTN < C'1 ≈ C'2 ≈ C'3
IRGS	< 0.0001	0.9835	< 0.0001	< 0.0001	< 0.0001	< 0.0001	0.9680	NTN ≈ C'1 < C'2 ≈ C'3
**Critical biological pathways in cancer**								
AKT	< 0.0001	0.5039	0.0006	< 0.0001	0.4897	0.2397	0.9552	NTN < C'2 ≈ C'3, NTN ≈ C'1 and C'1 ≈ C'2 ≈ C'3
β-Catenin	0.0976							NS
CIN	< 0.0001	0.5034	< 0.0001	< 0.0001	< 0.0001	< 0.0001	0.9992	NTN ≈ C'1 < C'2 ≈ C'3
E2F3	< 0.0001	0.9770	< 0.0001	0.4876	0.0004	0.9346	0.0007	NTN ≈ C'1 ≈ C'3 < C'2
EGFR	< 0.0001	0.0272	< 0.0001	< 0.0001	< 0.0001	< 0.0001	0.6211	NTN < C'1 < C'2 ≈ C'3
HOXA	< 0.0001	< 0.0001	0.0631	0.7829	0.0044	< 0.0001	0.0523	NTN ≈ C'2 ≈ C'3 < C'1
MITO/OXPHOS	0.1304							NS
MYC	< 0.0001	0.9606	< 0.0001	< 0.0001	< 0.0001	< 0.0001	0.6805	NTN ≈ C'1 < C'2 ≈ C'3
p53	< 0.0001	< 0.0001	< 0.0001	< 0.0001	< 0.0001	< 0.0001	0.5453	NTN > C'1 > C'2 ≈ C'3
PIK3CA	< 0.0001	0.3418	< 0.0001	< 0.0001	< 0.0001	0.0002	0.0008	NTN ≈ C'1 > C'3 > C'2
PNI	< 0.0001	0.6786	< 0.0001	0.4394	< 0.0001	0.2358	0.0042	NTN ≈ C'1 ≈ C'3 < C'2
PRL	< 0.0001	0.9976	< 0.0001	< 0.0001	0.0002	0.0144	0.5391	NTN ≈ C'1 > C'2 ≈ C'3
Proliferation	< 0.0001	0.3042	< 0.0001	< 0.0001	< 0.0001	< 0.0001	0.9761	NTN ≈ C'1 < C'2 ≈ C'3
PTEN loss	< 0.0001	0.9999	< 0.0001	< 0.0001	< 0.0001	< 0.0001	0.9763	NTN ≈ C'1 < C'2 ≈ C'3
RAS	< 0.0001	< 0.0001	0.0027	< 0.0001	0.1447	0.6591	0.0011	NTN < C'2 < C'3, NTN < C'1 ≈ C'2 and C'1 ≈ C'3
Reactive stroma	< 0.0001	0.3017	< 0.0001	< 0.0001	< 0.0001	< 0.0001	0.4652	NTN ≈ C'1 > C'2 ≈ C'3
SRC	0.0003	0.1305	0.9515	0.0023	0.1506	0.0003	0.1153	NTN ≈ C'1 < C'3, NTN ≈ C'1 ≈ C'2 and C'2 ≈ C'3
Stroma-CD10	< 0.0001	0.8668	< 0.0001	< 0.0001	< 0.0001	< 0.0001	0.9410	NTN ≈ C'1 > C'2 ≈ C'3
TGFβ	< 0.0001	< 0.0001	< 0.0001	< 0.0001	0.9616	0.5604	0.1722	NTN < C'1 ≈ C'2 ≈ C'3
VEGF	< 0.0001	< 0.0001	< 0.0001	< 0.0001	0.1496	0.7079	0.0016	NTN < C'3 < C'2, NTN < C'1 ≈ C'2 and C'1 ≈ C'3
Wound-response	< 0.0001	0.9446	< 0.0001	< 0.0001	< 0.0001	< 0.0001	0.9039	NTN ≈ C'1 < C'2 ≈ C'3
YAP1-WWTR1	NS							NS
**Prognosis**								
38-GES	< 0.0001	< 0.0001	< 0.0001	< 0.0001	< 0.0001	< 0.0001	0.2937	NTN < C'1 < C'2 ≈ C'3
70-GES	< 0.0001	< 0.0001	< 0.0001	< 0.0001	< 0.0001	< 0.0001	0.7985	NTN < C'1 < C'2 ≈ C'3
GGI	< 0.0001	0.0373	< 0.0001	< 0.0001	< 0.0001	< 0.0001	0.8691	NTN < C'1 < C'2 ≈ C'3

GES: gene-expression signature; NS: not significant (*P* > 0.05).
