# Supplementary material for: Identification of three subtypes of triple-negative breast cancer with potential therapeutic implications
Source: Breast Cancer Res. 2019 May 17;21:65. doi: 10.1186/s13058-019-1148-6 (PMC6525459; doi:10.1186/s13058-019-1148-6)

Additional file 27: Categorical GES distributions in function of external TNBC (C'1, C'2, C'3) and non-TNBC clusters (NTN). (A) CIT, **(B)** ER-negative and **(C)** PAM50.

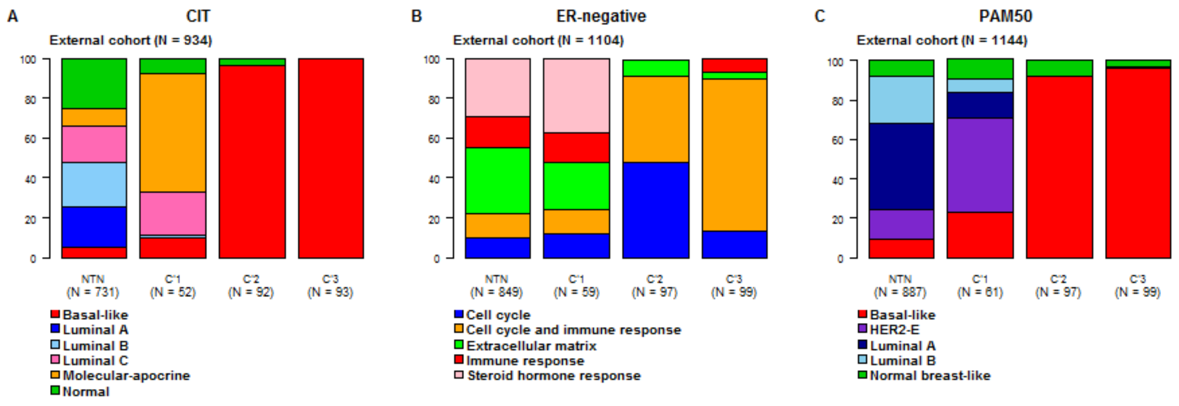

Supplement: Supplementary file 27 — Categorical GES distributions in function of external TNBC (C’1, C’2, C’3) and non-TNBC clusters (NTN). (A) CIT, (B) ER-negative and (C) PAM50. (PDF 138 kb) [file 13058_2019_1148_MOESM27_ESM.pdf]
