# Supplementary material for: Identification of three subtypes of triple-negative breast cancer with potential therapeutic implications
Source: Breast Cancer Res. 2019 May 17;21:65. doi: 10.1186/s13058-019-1148-6 (PMC6525459; doi:10.1186/s13058-019-1148-6)

Additional file 28: Molecular annotation of external cohort in function of TNBC clusters and non-TNBC. Radar plot showing the segregation of three clusters as a function of 47 continuous score GES. This figure is an illustration of Additional file 26. Each of the 47 radii represents a GES. Black circles represent significantly different levels of expression from low (smallest circle) to high (largest circle). Expression level of each cluster is represented on the corresponding circle as a dark grey (NTN), blue (C’1), red (C’2) or green (C’3) dot. Dots located on same circles correspond to clusters with not significantly different expressions. Dots located on different circles correspond to clusters with significantly different expressions. Dots located in between black circles correspond to a cluster with expression level not significantly different from clusters whose dots are located on both near circles.

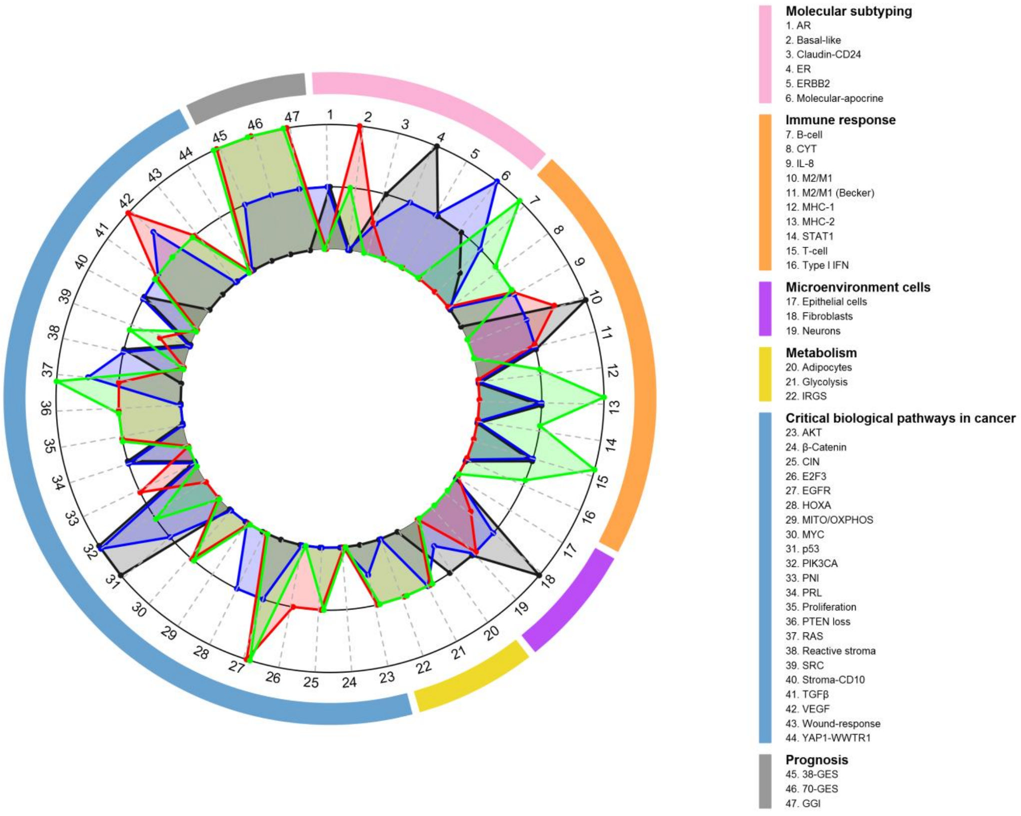

Supplement: Supplementary file 28 — Molecular annotation of external cohort in function of TNBC clusters and non-TNBC. Radar plot showing the segregation of three clusters as a function of 47 continuous score GES. This figure is an illustration of Additional file 26. Each of the 47 radii represents a GES. Black circles represent significantly different levels of expression from low (smallest circle) to high (largest circle). Expression level of each cluster is represented on the corresponding circle as a dark gray (NTN), blue (C’1), red (C’2) or green (C’3) dot. Dots located on same circles correspond to clusters with not significantly different expressions. Dots located on different circles correspond to clusters with significantly different expressions. Dots located in between black circles correspond to a cluster with expression level not significantly different from clusters whose dots are located on both near circles. (PDF 287 kb) [file 13058_2019_1148_MOESM28_ESM.pdf]
