# Supplementary material for: Identification of three subtypes of triple-negative breast cancer with potential therapeutic implications
Source: Breast Cancer Res. 2019 May 17;21:65. doi: 10.1186/s13058-019-1148-6 (PMC6525459; doi:10.1186/s13058-019-1148-6)
Supplement: Supplementary file 29 — Proposed therapeutic strategies in function of GES and robust subtyping results. (PDF 162 kb) [file 13058_2019_1148_MOESM29_ESM.pdf]

Additional file 29: Proposed therapeutic strategies in function of GES and robust subtyping results.

Subtyping result	GES name	Proposed therapeutic strategy	Reference
C1	AR	Antiandrogens	(S45)
	ERBB2	Dual anti-HER2 therapeutic approach	(S46, S47)
	Molecular apocrine	Antiandrogens	(S45)
	PIK3CA	PIK3 inhibitors + antiandrogens	(S48)
	PRL	Anti-prolactin	(S49)
C2 and C3	p53	p53 restoration	(S50)
	CIN	Centrosome amplification inhibition	(S51)
	EGFR	EGFR inhibitors	(S52)
	IRGS	Iron depletion	(S53)
	MYC	MYC pathway inhibition	(S54, S55)
	PTEN loss	PTEN loss restoration	(S56, S57)
C2	CIBERSORT	Immunosuppression reversion	(S58)
	E2F3	E2F3 inhibitors	(S59)
	PNI	Perineural invasion inhibitors	(S60)
	M2/M1	TAM-focused therapeutic strategies	(S61)
	Neurons/Axonogenesis	Antineurogenic therapies, nerve dependence inhibitors	(S62-S64)
	VEGF	Antiangiogenesis	(S65)
C3	B-cell	Stimulation of adaptive immune response	(S58)
	CIBERSORT	Stimulation of adaptive immune response	(S58)
	CYT	Stimulation of adaptive immune response	(S58)
	MHC-1	Stimulation of adaptive immune response	(S58)
	MHC-2	Stimulation of adaptive immune response	(S58)
	STAT1	Stimulation of adaptive immune response	(S58)
	T-cell	Stimulation of adaptive immune response	(S58)
	Type I IFN	Stimulation of adaptive immune response	(S58)

References

S45. Ni M, Chen Y, Lim E, Wimberly H, Bailey ST, Imai Y et al. Targeting androgen receptor in estrogen receptor-negative breast cancer. Cancer Cell. 2011;20:119-31.

S46. Konecny GE. Emerging strategies for the dual inhibition of HER2-positive breast cancer. Curr Opin Obstet Gynecol. 2013;25:55-65.

S47. Vranic S, Schmitt F, Sapino A, Costa JL, Reddy S, Castro M et al. Apocrine carcinoma of the breast: a comprehensive review. Histol Histopathol. 2013;28:1393-409.

S48. Lehmann BD, Bauer JA, Schafer JM, Pendleton CS, Tang L, Johnson KC et al. PIK3CA mutations in androgen receptor-positive triple negative breast cancer confer sensitivity to the combination of PI3K and androgen receptor inhibitors. Breast Cancer Res. 2014;16:406.

S49. Kelly MP, Hickey C, Makonnen S, Coetzee S, Jalal S, Wang Y et al. Preclinical activity of the novel anti-prolactin receptor (PRLR) antibody-drug conjugate REGN2878-DM1 in PRLR-positive breast cancers. Mol Cancer Ther. 2017;16:1299-311.

S50. Zawacka-Pankau J, Selivanova G. Pharmacological reactivation of p53 as a strategy to treat cancer. J Intern Med. 2015;277:248-59.

S51. Leontovich AA, Salisbury JL, Veroux M, Tallarita T, Billadeau D, McCubrey J et al. Inhibition of Cdk2 activity decreases Aurora-A kinase centrosomal localization and prevents centrosome amplification in breast cancer cells. Oncol Rep. 2013;29:1785-88.

S52. Nakai K, Hung MC, Yamaguchi H. A perspective on anti-EGFR therapies targeting triple-negative breast cancer. Am J Cancer Res. 2016;6:1609-23.

S53. Torti SV, Torti FM. Cellular iron metabolism in prognosis and therapy of breast cancer. Crit Rev Oncog. 2013;18:435-48.

S54. Horiuchi D, Camarda R, Zhou AY, Yau C, Momcilovic O, Balakrishnan S et al. PIM1 kinase inhibition as a targeted therapy against triple-negative breast tumors with elevated MYC expression. Nat Med. 2016;22:1321-9.

S55. Fallah Y, Brundage J, Allegakoen P, Shajahan-Haq AN. MYC-driven pathways in breast cancer subtypes. Biomolecules. 2017;7(3).
S56. Ebbesen SH, Scaltriti M, Bialucha CU, Morse N, Kastenhuber ER, Wen HY et al. Pten loss promotes MAPK pathway dependency in HER2/neu breast carcinomas. Proc Natl Acad Sci USA. 2016;113:3030-5.
S57. Dillon LM, Miller TW. Therapeutic targeting of cancers with loss of PTEN function. Curr Drug Targets. 2014;15:65-79.
S58. Di Caro G, Castino GF, Bergomas F, Cortese N, Chiriva-Internati M, Grizzi F et al. Tertiary lymphoid tissue in the tumor microenvironment: from its occurrence to immunotherapeutic implications. Int Rev Immunol. 2015;34:123-33.
S59. Lee M, Oprea-Ilies G, Saavedra HI. Silencing of E2F3 suppresses tumor growth of Her2+ breast cancer cells by restricting mitosis. Oncotarget. 2015;6:37316-34.
S60. Amit M, Na'ara S, Gil Z. Mechanisms of cancer dissemination along nerves. Nat Rev Cancer. 2016;16:399-408.
S61. Mantovani A, Marchesi F, Malesci A, Laghi L, Allavena P. Tumour-associated macrophages as treatment targets in oncology. Nat Rev Clin Oncol. 2017;14:399-416.
S62. Magnon C, Hall SJ, Lin J, Xue X, Gerber L, Freedland SJ et al. Autonomic nerve development contributes to prostate progression. Science. 2013;341:1236361.
S63. Jobling P, Pundavela J, Oliveira SM, Roselli S, Walker MM, Hondermarck H. Nerve-cancer cell cross-talk: a novel promoter of tumor progression. Cancer Res. 2015;75:1777-81.
S64. Boilly B, Faulkner S, Jobling P, Hondermarck H. Nerve dependence: from regeneration to cancer. Cancer Cell. 2017;31:342-54.
S65. Ribatti D, Nico B, Ruggieri S, Tamma R, Simone G, Mangia A. Angiogenesis and antiangiogenesis in triple-negative breast cancer. Transl Oncol. 2016;9:453-7.
